# Spotlight onto surfactant–steam–bitumen interfacial behavior via molecular dynamics simulation

**DOI:** 10.1038/s41598-021-98633-1

**Published:** 2021-10-04

**Authors:** Mohammadali Ahmadi, Zhangxin Chen

**Affiliations:** Department of Chemical and Petroleum Engineering, Schulich School of Engineering, University of Calgary, Calgary, AB T2N1T4 Canada

**Keywords:** Crude oil, Petrol, Chemical engineering

## Abstract

Heavy oil and bitumen play a vital role in the global energy supply, and to unlock such resources, thermal methods, e.g., steam injection, are applied. To improve the performance of these methods, different additives, such as air, solvents, and chemicals, can be used. As a subset of chemicals, surfactants are one of the potential additives for steam-based bitumen recovery methods. Molecular interactions between surfactant/steam/bitumen have not been addressed in the literature. This paper investigates molecular interactions between anionic surfactants, steam, and bitumen in high-temperature and high-pressure conditions. For this purpose, a real Athabasca oil sand composition is employed to assess the phase behavior of surfactant/steam/bitumen under in-situ steam-based bitumen recovery. Two different asphaltene architectures, archipelago and Island, are used to examine the effect of asphaltene type on bitumen's interfacial behavior. The influence of having sulfur heteroatoms in a resin structure and a benzene ring's effect in an anionic surfactant structure on surfactant–steam–bitumen interactions are investigated systematically. The outputs are supported by different analyses, including radial distribution functions (RDFs), mean squared displacement (MSD), radius of gyration, self-diffusion coefficient, solvent accessible surface area (SASA), interfacial thickness, and interaction energies. According to MD outputs, adding surfactant molecules to the steam phase improved the interaction energy between steam and bitumen. Moreover, surfactants can significantly improve steam emulsification capability by decreasing the interfacial tension (IFT) between bitumen and the steam phase. Asphaltene architecture has a considerable effect on the interfacial behavior in such systems. This study provides a better and more in-depth understanding of surfactant–steam–bitumen systems and spotlights the interactions between bitumen fractions and surfactant molecules under thermal recovery conditions.

## Introduction

A considerable portion of the global oil reserves is comprised of oil sands and bitumen, such as those in Alberta, Canada^1^. To unlock these reserves, in-situ steam methods have been applied. However, such methods suffer from abundant greenhouse gas (GHG) emissions, high-risk operational barriers, high capital costs^2,3^, and environmental footprints^3–6^. These issues constrain the use of steam injection for improving oil sands and bitumen recovery^3,7–10^. Hence, there is an urgent need to develop a new type of technology to tackle these issues in recovery of oil sands and bitumen. Among different available options, surface active agents are potential solutions to improve their recovery performance by altering the physicochemical characteristics of oil sands and bitumen^11–13^. Using surfactants as an additive to steam showed a promising performance in both pilot and field scales^14–22^. However, many unanswered fundamental questions remain; how surfactants interact with bitumen in high pressure and temperature conditions and how emulsification of oil at high temperature behaves, for example. These new challenges must be properly addressed before the application of surfactants as an effective additive to steam^23–38^.

Molecular Dynamics (MD) simulation offers a helpful tool to address the fundamental question regarding oil sands and surfactants' behavior under high temperatures and high-pressure conditions. A MD simulation approach has been successfully implemented in a wide range of applications from drug delivery^39–41^ to adsorption of hydrocarbons on minerals^42–45^ and flow behavior of fluids inside nanopores^46–48^. Below most of the related works will be given on systems containing oil and chemicals. Jang et al.^49^ investigated the effect of an anionic surfactant architecture, Alkyl Benzene Sulfonate, on interfacial properties between decane and water. They employed different scenarios to determine the best location for attaching a benzene sulfonate group, which resulted in lower interface formation energy, and also studied interfacial thickness and found a relationship between interfacial tension (IFT) and interfacial thickness^49^.

Wu et al.^50^ employed the MD simulation approach to evaluate the adsorption and desorption behavior of Athabasca oil sands fractions onto a quartz surface at different temperatures. They examined three different temperatures, 298, 398, and 498 K, to conclude an impact of temperature on heavy oil fractions' aggregation behavior and their sorption trend onto the quartz surface. According to their MD simulation results, they revealed that an increase in temperature significantly increased the diffusion coefficient of heavy oil fractions, and it also had a meaningful effect on the sorption behavior of these fractions.

Tang et al.^51^ studied a process of oil recovery during surfactant flooding in nanoporous quartzes using two different types of surfactants, including cationic, dodecyl tri-methyl-ammonium bromide, and anionic, Dodecyl Benzene Sulfonate (SDBS). According to their results they gained from MD simulation, the headgroup's tendency to form hydrogen bonds with water as an anionic surfactant was higher than the cationic one. This means that a detachment of oil from a sand surface in a case of anionic surfactant flooding was quicker than cationic surfactant injection.

Iwase et al.^52^ developed a digital oil model to simulate an oil recovery process using carbon dioxide, methane, and several solvents. They carried out a series of numerical experiments in a wide range of pressures and temperatures and validated their MD outputs with experimental data. They revealed that methane has excellent potential for a viscosity reduction compared to other solvents they used based on MD simulations.

Lv et al.^53^ employed MD simulation to model an effect of copolymer on heavy oil’s viscosity. They focused on a possible bond between heavy oil fractions, especially asphaltenes, and the copolymer viscosity reducer. Their results revealed that the number of hydrogen bonds in asphaltenes decreases when a system's copolymer content increases. Consequently, adding copolymers could significantly reduce the viscosity of a heavy oil sample.

Jian et al.^54^ applied MD simulation to understand how asphaltene molecules interact at an interface of water and toluene at different temperatures. They ran several different scenarios comprising different simulation box sizes, asphaltene molecules, pressures, and temperatures. They concluded that both temperature and asphaltene concentration could play a significant role in the behavior of asphaltene molecules at a water–toluene interface; this behavior can vary from a solute-like agent to surfactant-like molecules. Song et al.^55^ carried out MD simulation on a mixture of asphaltene and resin in the presence of an anionic surfactant, i.e., Sodium Dodecyl Sulfate. Based on their results, adding surfactants facilitate a viscosity reduction in heavy oils due to a weak interaction between resins and asphaltene molecules.

Ahmadi and Chen^56^ carried out MD simulation in a preliminary study on the interfacial behavior between asphaltenes and surfactants in an aqueous solution. Both archipelago and island architectures were used in their work to investigate an effect of an asphaltene structure. Their outputs revealed that anionic surfactant interacted with asphaltenes more than other surfactant types. They also investigated the behavior of non-anionic and anionic surfactants in hydrocarbon solvents as asphaltene dispersants^57^. Their results showed that one of the main factors which affected the efficiency of surfactant dispersant was an asphaltene molecular size. By increasing the asphaltene molecular size, the performance of surfactant dispersant was decreased. They also performed a comprehensive study on the effect of surfactant’s benzene ring on an interaction between asphaltene and surfactants in aqueous solutions. MD outputs revealed that a benzene ring improved the van der Waals interactions between surfactant and asphaltene because of having more π–π interactions. It is worth mentioning that the main contributor of π–π interactions was face-to-edge interactions between aromatic fused sheets on asphaltene and benzene rings on a surfactant^58^. Table 1 reports a summary of MD works carried out on systems of oil with/without chemicals. This table summarizes the remarkable findings, the representative composition of the oil phase, a type of force field, thermodynamic conditions of MD simulation, simulation time, and chemical composition in these works.

**Table 1:** Summary of MD studies for oil systems with/without chemical.

Year	Researcher	Temperature (K)	Pressure (MPa)	Force field	Chemical	Oil	Remark
2019	Su et al.^59^	300	0.1	GROMOS 53a6^60^	Sodium dodecyl sulfate (SDS)	Two types of Asphaltene and Resins	They investigated the impact of the presence of SDS on the aggregation behavior of noncarboxyle and carboxylic asphaltenes. They figured out SDS could emulsify the oil droplet and then binds with the oil–water surface in carboxylic asphaltene are stronger than non-carboxylic ones
2018	Meng et al.^61^	300	–	CHARMM36^62^	–	A mixture of 12 Resin, 6 Asphaltene, 18 Aromatics, and 21 Saturates	The process of heavy oil adsorption onto the silica surface was studied using MD simulation. Based on their outcomes, changing the grooves' size and shape on the silica surface changed the amount of adsorbed heavy oil onto the silica surface. Also, the presence of water molecules could decrease the adsorption of heavy oil onto the silica surface
2018	Song et al.^55^	300	0.1	GROMOS 53a6^60^	Sodium dodecyl sulfate	Asphaltene and Resin	De-emulsification of the asphaltene-water system was investigated through a molecular simulation study. According to their results, SDS could increase the hydrophilic area on the water-asphaltene surface and de-emulsify the oil droplet in the water phase
2018	Khalaf and Mansoori^63^	300	0.1	Optimized potentials for liquid simulations: all atom (OPLS-AA)^64,65^	–	Three different Asphaltene	The onset of asphaltene aggregation in three synthetic oil samples, comprised of n-heptane and o-xylene, was evaluated using molecular simulation. Based on their outcomes, face-to-face staking occurred in high concentration n-heptane. Also, they found that the hydrogen bond could stabilize the aggregation
2018	Jian et al.^54^	300–473	10–20	GROMOS96^66^	Violanthrone-79	Toluene	The effect of asphaltene on the interfacial behavior between toluene and water was examined. Based on their simulation outputs, increasing the temperature resulted in decreasing the asphaltene molecules' solubility and changing the nature of asphaltene from the surface-active agent to the solute
2017	Mao et al.^67^	323	–	Condensed-phase optimized molecular potential for atomistic simulation studies (COMPASS)	Quadripolymers	Heavy component extracted from China Oil Fields	The molecular simulation was employed to evaluate the mechanisms behind the viscosity reduction of heavy oil in the presence of oil-soluble copolymer. They revealed that the presence of copolymer could change the structure of the agglomerates of asphaltene and resins, and consequently, it could improve the flow characteristic of heavy oil
2016	Li et al.^68^	348	–	COMPASS	Cetyltrimethyl ammonium bromide, dodecyl betaine, sodium dodecyl benzene sulfonate, fatty alcohol-poly-oxyethylene ether	Dodecane	The oil detachment process from different surfaces, including calcite, dolomite, silica, and siderite, was evaluated using MD simulation in the presence of four different types of surfactants. According to the results gained from MD, they revealed that nonionic and amphoteric surfactants could detach oil from the calcite surface quicker than anionic and cationic surfactants. However, the story is different for the case of the dolomite surface
2015	Wu et al.^69^	298	–	COMPASS	–	2 Asphaltenes, 6 Aromatics, 4 Resins, 7 Saturates	The impact of the organic materials on the adsorption of heavy oil fractions onto the quartz surface was systematically studied via MD simulation. The MD results revealed that the presence of fulvic and humic acids could improve heavy crude oil fractions' adsorption
2014	Zolghadr et al.^70^	343.15	0.1	OPLS-AA^65,66^	SDS	Heptane	The interfacial behavior of the system containing Heptane/Water and SDS was studied via MD simulations. There was a good agreement between the IFT measured values and simulated ones. SDS could diffuse to the bulk of heptane at a high rate
2010	Gang et al.^71^	293	–	MMFF94	Surfactin	Octane	The behavior of surfactin at the Octane/water interface was evaluated using MD simulation
2009	Lu et al.^72^	373	–	COMPASS	Dodecyl benzene sulfonate, sodium hexadecyl sulfonate, rhamnolipid	Octane	The effect of surfactant on the wettability alteration of the quartz crystal surface was evaluated using molecular modeling. They found that adding a surfactant to the oil-quartz system could reduce the quartz surface's hydrophobicity, resulting in changing the surface's wettability toward water-wet. The surfactant could also increase the distance between the oil molecules and the quartz surface, which is another proof of wettability alteration in such a system

To the best of our knowledge, no work can be found in the literature to study molecular interfacial interactions between surfactant, steam, and bitumen. This paper's main objective is to establish a fundamental theoretical understanding of bitumen behavior under a steam/chemical co-injection process and study how they depend on pressure, temperature, and other related parameters. For this purpose, we employed two different asphaltene architectures and two resin structures to study the effect of an asphaltene architecture and resin’s sulfur heteroatoms on the interfacial behavior of bitumen droplets at a steam–surfactant interface. Two anionic surfactants, including SDBS and SDS, which are soluble in water, were also used to compare the sulfate and sulfonate functional groups on a steam–bitumen interface's interfacial properties.

## Methodology

### System initialization

A Materials Studio^73^ software package has been used to carry out MD simulation processes, and the COMPASS force field was used. The COMPASS force field has successfully been applied to different systems to predict internal properties and cover a wide range of materials, including heavy fractions of oil, acids, diesel, and quartz surfaces^50,69,74–77^.

The convergence criterion of geometry optimization was set to 1000 kJ mol^−1^ nm^−1^ to optimize the system's geometry. The periodic boundary conditions were utilized in the entire simulation box^59,78^. The time step in all simulation runs was one fs, and the system temperature was set to 498 K to have a thermodynamic condition close to the real condition of in-situ steam-based bitumen recovery with chemical additives^22,79,80^. A Nose–Hoover–Langevin (NHL) thermostat^81–83^ and a Brendensen barostat^84^ were used to control the temperature of a system in each simulation. These parameters have extensively been validated and effectively utilized for studying the molecular behavior of heavy oil and bitumen^85–87^. The Ewald summation method was implemented to capture Coulomb interactions during our MD simulation, and a cut-off distance of 12 A was employed to evaluate van der Waals interactions by an atom-based summation approach^59,88,89^.

### Simulation system

The sections below provide an insight into the system, including oil samples and surfactant solutions, for performing MD simulations.

#### Oil samples and surfactant solutions

According to the Saturate, Aromatic, Resin, and Asphaltene (SARA) analysis reported for Athabasca oil sands samples, a mass ratio of 15:30:35:20, respectively, was employed to construct a bitumen sample^69,90^. The bitumen samples were formed by adding two types of asphaltene (Island and archipelago), two types of resin (A and B), saturates, and aromatics. According to the structure of the asphaltene, there are two different configurations: Archipelago and Island. The archipelago architecture comprises several aromatic sheets attached through alkyl chains^91^. The Island (continental) architecture is a centered condensed aromatic sheet inside the asphaltene molecules attached to several alkyl chains^92–94^. Six asphaltene molecules, eight aromatic molecules, eighteen resin molecules, and nine saturate molecules were randomly placed in a 6 × 6 × 6 nm simulation box. It is worth highlighting that the asphaltene stability index, the ratio of asphaltene + saturates to aromatics + resins^95^, of a bitumen sample in this paper is almost 0.54, which means that the asphaltene molecules are stable in the oil phase^96,97^. Then geometry optimization was applied to the simulation box, and an Isothermal–isobaric (NPT) ensemble at 5 MPa and 498 K followed to gain a reasonable density at a typical oil sands reservoir pressure under a steam-assisted gravity drainage (SAGD) process. Figure S1 illustrates the molecular structures of the asphaltene, saturate, resin, aromatic, and anionic surfactant molecules used in this study^50,55,58,98,99^. To create a surfactant solution, 4000 water molecules and four surfactant molecules were placed into a 6 × 6 × 12 nm simulation box.

#### Simulation box

**Figure 1:**
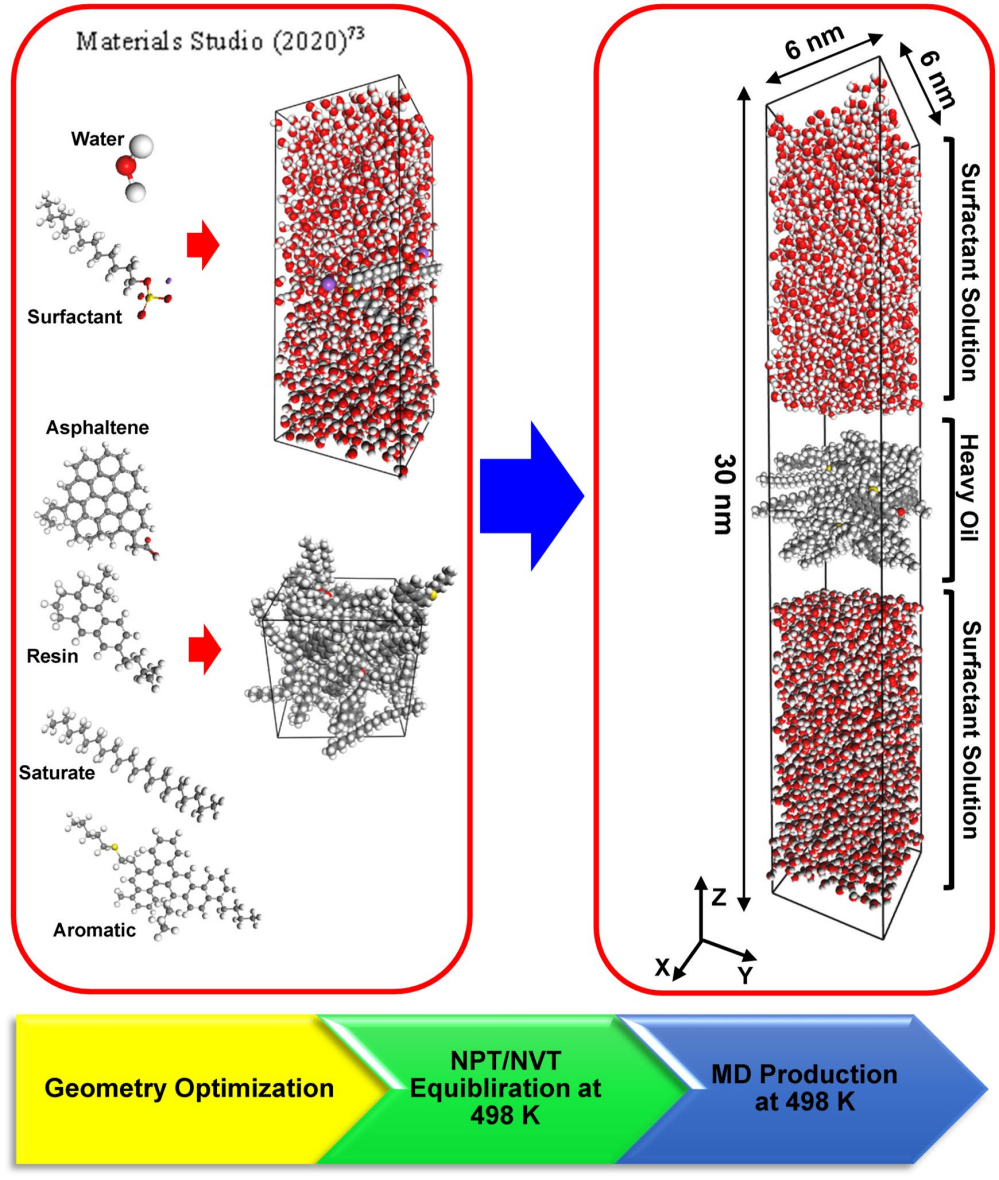
Schematic of MD simulation workflow.

**Figure 2:**
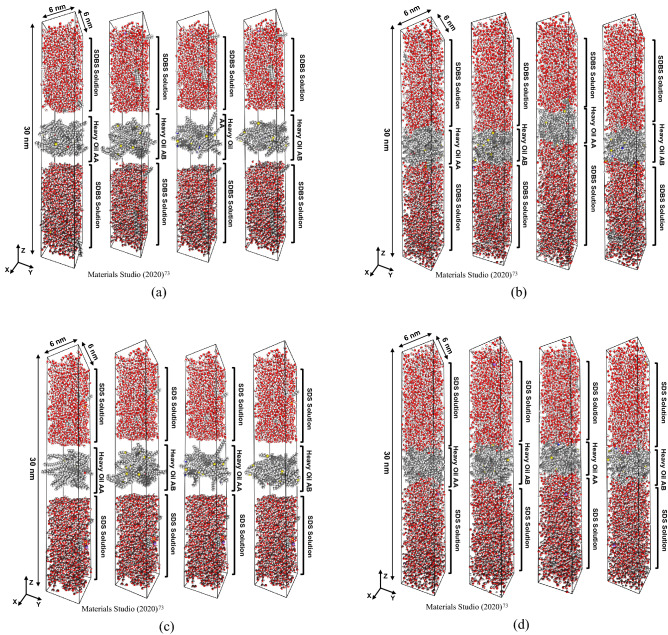
Snapshots of the initial and final configurations of simulation boxes: (**a**) SDBS initial configuration, (**b**) SDBS final configuration, (**c**) SDS initial configuration, and (**d**) SDS final configuration.

Figure 1 depicts a schematic of the workflow employed to perform MD simulation in surfactant/steam/bitumen systems. As shown in Fig. 1, a bitumen sample was placed between two surfactant solutions to create the simulation box. The details of the systems under study are reported in Table 2. Table 2 illustrates the system ID, types of surfactants, resins, and asphaltenes. As demonstrated in Fig. 1, after creating a simulation box, the optimization process was followed to minimize the system's energy. The next step is equilibrating the simulation box, which comprises a 1000 ps NPT ensemble and a 1000 ps Canonical ensemble (NVT). Using the NPT ensemble helps us to achieve a reasonable density for the system at our desired temperature. Finally, after equilibrating the system, the simulation box is ready to perform MD simulation for 50,000,000 time steps. Figure 2 shows the initial and equilibrated configurations of the simulation boxes for both SDS and SDBS surfactants.

**Table 2:** Details of MD simulation systems.

System ID	Asphaltene type	Resin type	Surfactant
SB-AA	C_40_H_30_O_2_	C_23_H_30_	SDBS
SB-AB	C_40_H_30_O_2_	C_22_H_30_S	SDBS
SB-BA	C_44_H_40_N_2_OS	C_23_H_30_	SDBS
SB-BB	C_44_H_40_N_2_OS	C_22_H_30_S	SDBS
S-AA	C_40_H_30_O_2_	C_23_H_30_	SDS
S-AB	C_40_H_30_O_2_	C_22_H_30_S	SDS
S-BA	C_44_H_40_N_2_OS	C_23_H_30_	SDS
S-BB	C_44_H_40_N_2_OS	C_22_H_30_S	SDS
W-AA	C_40_H_30_O_2_	C_23_H_30_	–
W-AB	C_40_H_30_O_2_	C_22_H_30_S	–
W-BA	C_44_H_40_N_2_OS	C_23_H_30_	–
W-BB	C_44_H_40_N_2_OS	C_22_H_30_S	–

## Results and discussion

The sections below describe the results of MD simulation in the above surfactant/steam/bitumen systems in high-temperature and high-pressure conditions.

### Equilibrium

As explained in “System initialization”, the first step for initializing MD simulation is performing geometry optimization to assure we have a system at its minimum energy configuration. Based on our cases’ nature, we performed 1000 ps NPT followed by 1000 ps NVT ensembles to achieve a reasonable density. Table 3 reports the systems' energy components, including kinetic energy, potential energy, and non-bond energy, at equilibrium conditions at 498 K.

**Table 3:** Energy components of the system after the equilibration stage at 498 K.

System ID	Total energy (kcal/mol)	Kinetic energy (kcal/mol)	Non-bond energy (kcal/mol)	Potential energy (kcal/mol)
SB-AA	13,879.827	22,705.495	− 29,589.699	− 8825.667
SB-AB	14,229.870	22,607.148	− 29,249.181	− 8377.278
S-AA	13,497.197	22,694.385	− 30,207.841	− 9197.188
S-AB	12,971.638	22,470.738	− 29,919.008	− 9499.100
SB-BA	12,790.881	22,497.916	− 28,937.240	− 9707.035
SB-BB	12,446.302	22,536.757	− 28,905.633	− 10,090.455
S-BA	12,029.605	22,419.086	− 29,504.197	− 10,389.481
S-BB	11,824.646	22,481.829	− 29,570.826	− 10,657.183
W-AA	14,484.753	21,982.889	− 28,131.083	− 7498.136
W-AB	14,384.466	21,839.394	− 27,977.506	− 7454.928
W-BA	13,029.544	21,927.125	− 27,562.753	− 8897.581
W-BB	13,351.203	22,069.955	− 27,673.800	− 8718.752

### Radial distribution function (RDF)

A radial distribution function (RDF) is defined as the ratio of the density of a particular atom in the distance of *r* to the bulk density. In other words, the variation of density of a particular atom with a change in distance with reference molecules over the bulk density represents RDF. So, it can be used to demonstrate a density distribution around a given molecule, and it is mathematically expressed as follows:

1 $${g}_{ab}\left(r\right)=\frac{V}{{N}_{a}{N}_{b}}\left(\sum_{i=1}^{{N}_{a}}\frac{{n}_{i}b(r)}{4\pi {r}^{2}\Delta r}\right)$$

where *N*_*a*_ and *N*_*b*_ represent the total numbers of atoms *a* and *b*, respectively, *V* stands for the simulation box’s volume, and *n*_*i*_*b*(*r*) denotes the number of atom *b* at the radial distance of *r* from atom *a*.

**Figure 3:**
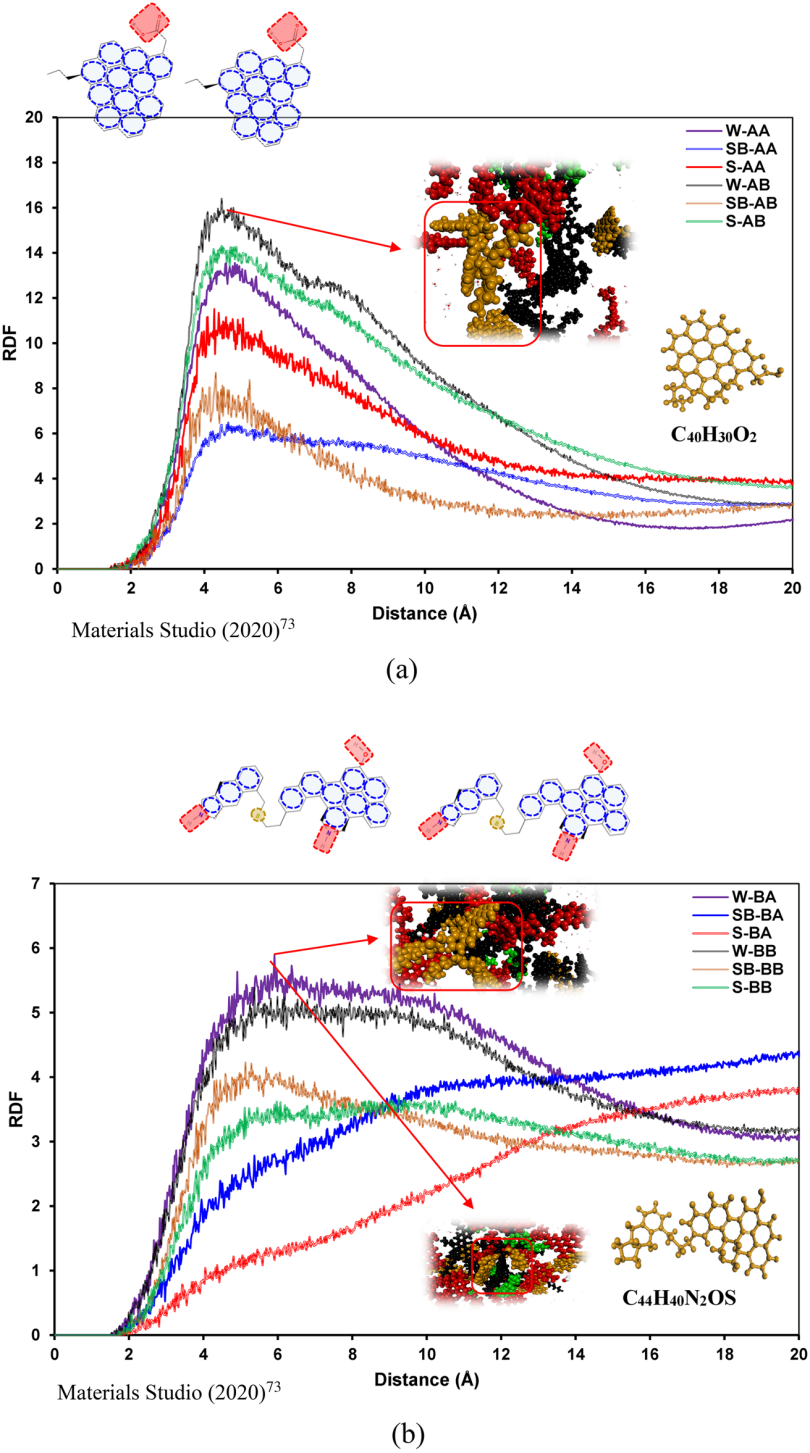
RDFs of the asphaltene molecule pairs containing (**a**) C_40_H_30_O_2_ and (**b**) C_44_H_40_N_2_OS.

To have transparent snapshots during a visualization process, we applied unique colors for every type of molecule. In this regard, surfactants are denoted by blue, saturates are represented by green, resins are depicted in red, aromatics are black, and asphaltenes are goldish brown. Figure 3 demonstrates the RDF plots of asphaltene pair molecules for different systems. As illustrated in Fig. 3a, in the case of having asphaltene A (C_40_H_30_O_2_) in an oil sample, SDBS has similar trends for both resins; however, RDF_max_ in the case of resin C_23_H_30_ (6.01) is slightly lower than C_23_H_30_S (7.14). The reason for observing this slight difference is few hydrogen bonds between SDBS and asphaltene molecule (see Fig. 5a). This behavior is also observed for system S-AA, which has a lower spike compared to system S-AB containing asphaltene A (C_40_H_30_O_2_) and resin B (C_22_H_30_S), as shown in Fig. 3a.

Furthermore, the existence of sulfur in the resin structure has a meaningful effect on the asphaltene–asphaltene interactions in the case of the island asphaltene structure (see Fig. 3a). The sulfur atom in the resin structure resulted in a considerable increase in RDF_max_, which means that the macromolecular structure's size between island asphaltene molecules is meaningfully increased. In other words, the aggregation tendency of island asphaltene molecules in the presence of resin B (C_22_H_30_S) is higher than the cases with resin A.

In the case of having archipelago asphaltene (C_44_H_40_N_2_OS) and resin B (C_23_H_30_S), both SDS and SDBS have a similar RDF plot, which means that the asphaltene aggregation trend in the presence of SDS and SDBS is quite similar (see Fig. 3b). However, in this case, SDS has a lower RDF in comparison with SDBS, which reveals a slightly smaller asphaltene aggregate size. As shown in Fig. 3b, in both S-BA and SB-BA systems, no short-range macro-molecular structure for asphaltene pair molecules was observed. In other words, asphaltene molecules do not tend to create a nano-cluster in the presence of SDS/SDBS and resin A (C_22_H_30_). It is worth highlighting that the archipelago architecture was barely observed and reported in the literature. Due to the archipelago asphaltenes' structure, analyzing RDF plots without visualization may mislead to a wrong conclusion. Hence, some snapshots of the configurations are embedded into the RDF plots. As illustrated in Fig. 3b, one of the main reasons for observing a peak in a RDF plot of an asphaltene pair is bending the middle chain of an archipelago asphaltene molecule, which resulted in π–π interactions between left and right fused aromatic sheets.

**Figure 4:**
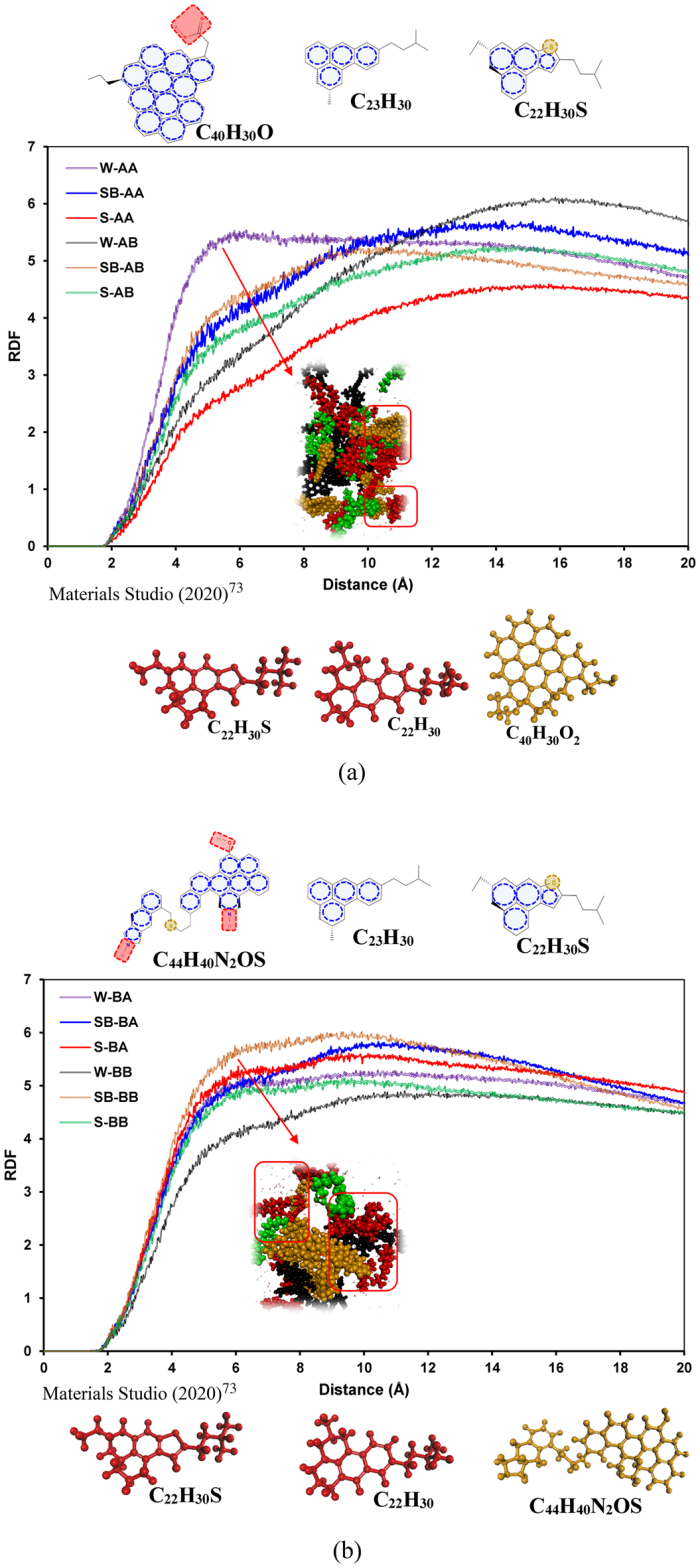
RDFs of the asphaltene–resin molecule pairs containing (**a**) C_40_H_30_O_2_ and (**b**) C_44_H_40_N_2_OS.

Figure 4 shows the RDF plots of asphaltene–resin pair molecules for all systems. As shown in Fig. 4a and b, the RDF plots for asphaltene–resin pairs have similar trends with the exception system S-AB. It means that resin B (C_22_H_30_S) actively interacts with island asphaltene in the presence of SDS (see Fig. 4a). As depicted in Fig. 4b, the RDF trends for the archipelago asphaltene and resin pairs are pretty similar; however, both resins in the presence of SDBS surfactant have higher RDF compared to systems with SDS surfactant.

**Figure 5:**
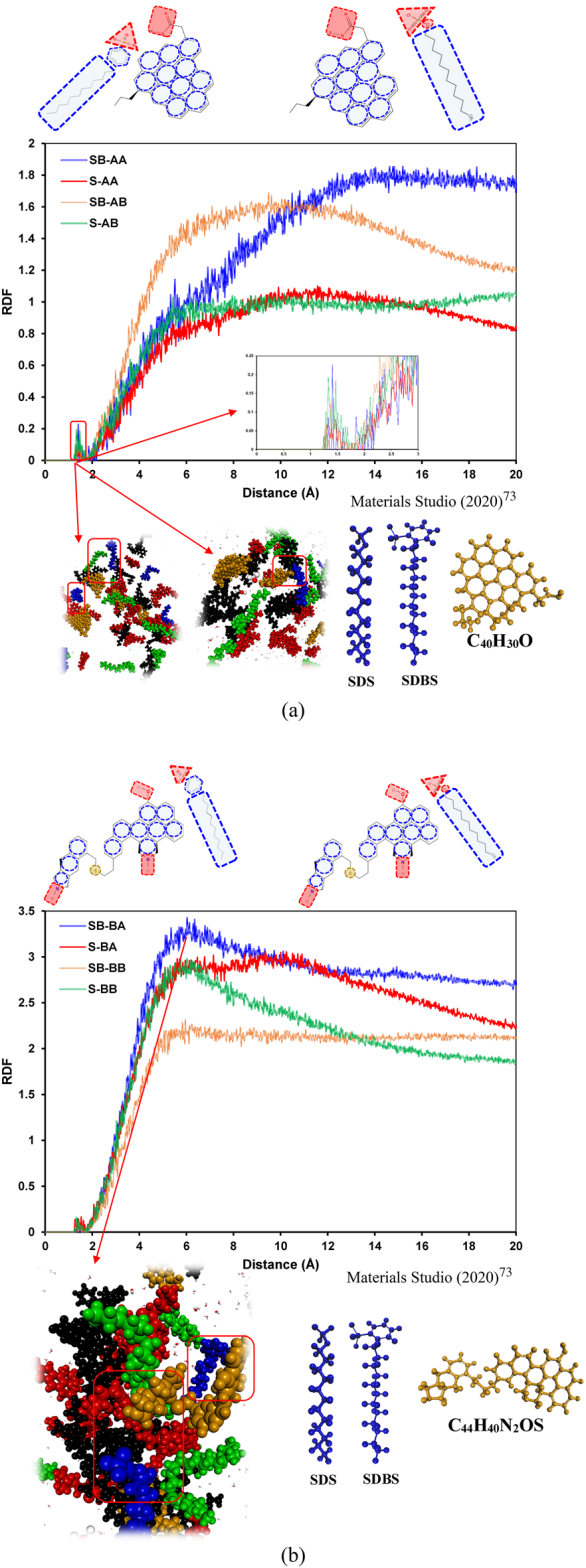
RDFs of the asphaltene–surfactant molecule pairs containing (**a**) C_40_H_30_O_2_ and (**b**) C_44_H_40_N_2_OS.

Figure 5 depicts the RDF curves of asphaltene–surfactant pair molecules for different systems. As shown in Fig. 5a, small spikes around a distance of 1.5 Å from asphaltene molecules for both SDS and SDBS were observed, which revealed hydrogen bonding between surfactants and asphaltenes. For short distance (less than 2Å), SDBS interacts better in a case of resin A (C_22_H_30_). Based on the RDF plots, SDBS has a greater interaction with island asphaltene molecules in the presence of resin B (C_22_H_30_S); conversely, the lowest RDF plot for asphaltene–island pair molecules belongs to the SB-AA system. It means that the presence of sulfur in the resin structure significantly affects the interactions between asphaltene–surfactant molecules. For systems S-AA and S-AB, sulfur in the resin structure resulted in lower interactions between SDS and island asphaltene molecules.

For systems containing archipelago asphaltenes, greater interaction between surfactant and archipelago was observed in the case of having SDS and resin A (C_23_H_30_); however, the RDF plot of SDS and archipelago asphaltenes has the lowest RDF in the presence of resin (C_22_H_30_S). It means that in the case of archipelago asphaltenes, the interaction between SDS and asphaltenes is drastically decreased if we have sulfur hetero-atoms in the resin structure. On the other hand, the interactions between SDBS and archipelago asphaltenes are increased in the case of having resin B (C_22_H_30_S) (see Fig. 5b). The same as for island asphaltenes, there are minor spikes around distance 1.5 Å from asphaltene molecules for both SDS and SDBS, which shows the hydrogen bonding between asphaltene and surfactants.

**Figure 6:**
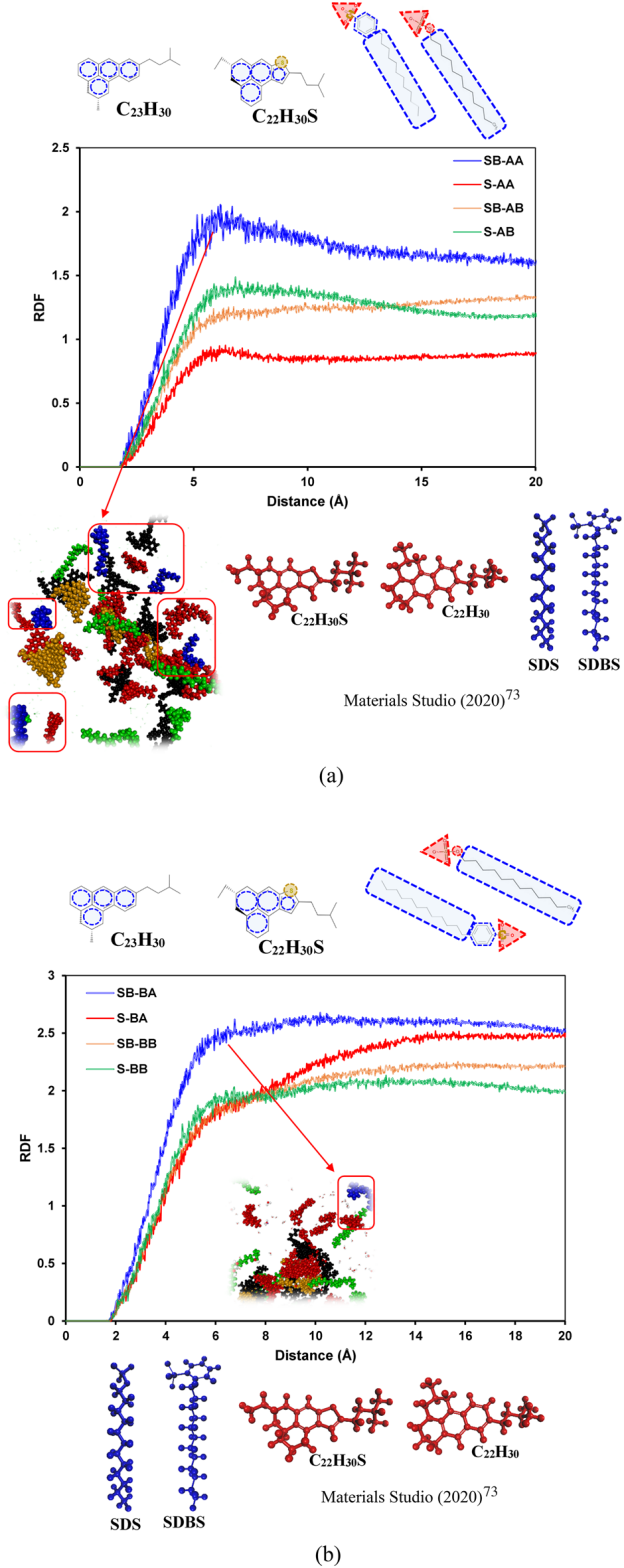
RDFs of the resin–surfactant molecule pairs containing (**a**) C_40_H_30_O_2_ and (**b**) C_44_H_40_N_2_OS.

Figure 6 illustrates the RDF plots of surfactant–resin pair molecules for all systems. As illustrated in Fig. 6a, resin A (C_22_H_30_) has the highest RDF with SDBS compared to SDS. On the other hand, adding sulfur to resin changed this trend significantly and resulted in having the same trend between resin B (C_22_H_30_S) and SDS/SDBS pairs. Similar behavior was observed for systems containing archipelago asphaltenes (see Fig. 6b). The possible cause for having such a trend between resin and surfactant is a sulfur heteroatom and its position in a resin molecule. Having sulfur in the resin structure slightly increases a resin molecule's polarity in the case of a similar molecular structure^100^. The primary interaction between surfactants and resins in our cases is driven by hydrophobic and π–π (between SDBS’s benzene ring and aromatic rings on resins) interactions. That is why we observed a lower RDF plot for resin B (C_22_H_30_S) and surfactants.

**Figure 7:**
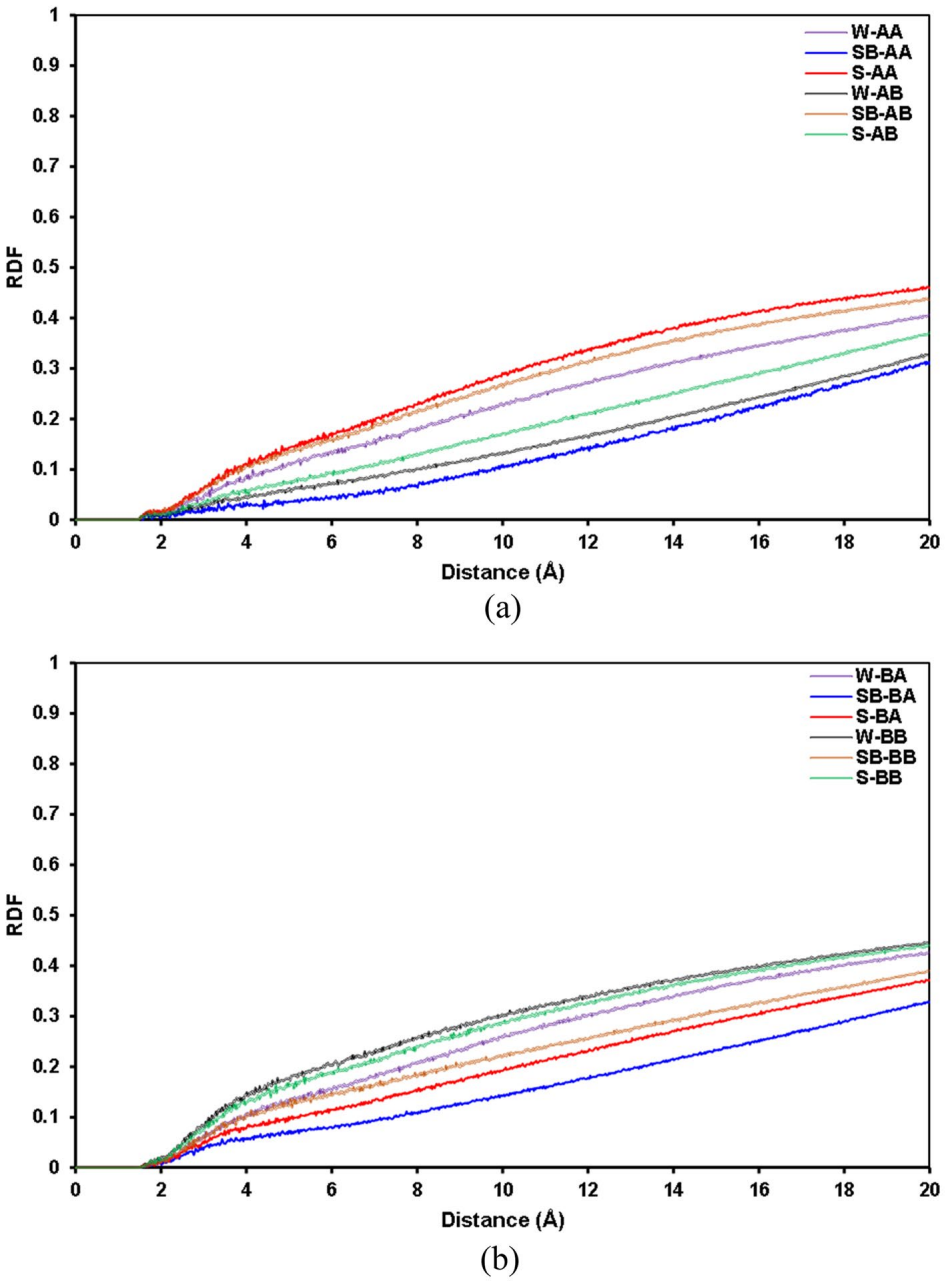
RDFs of the asphaltene–water molecule pairs containing (**a**) C_40_H_30_O_2_ and (**b**) C_44_H_40_N_2_OS.

Figure 7 depicts the RDF curves of asphaltene–water pair molecules for different systems. As depicted in Fig. 7a and b, there is no spike on the RDF plots, which means there is no nano-cluster or macro-molecular structure between asphaltene and water pairs. Moreover, the RDF trends are similar; however, for both archipelago and island architectures, SB-AA and SB-BA systems have the lowest RDF compared to other systems. It means that the interaction between asphaltene and water in SB-AA and SB-BA systems is slightly lower than that in the rest of the systems.

**Figure 8:**
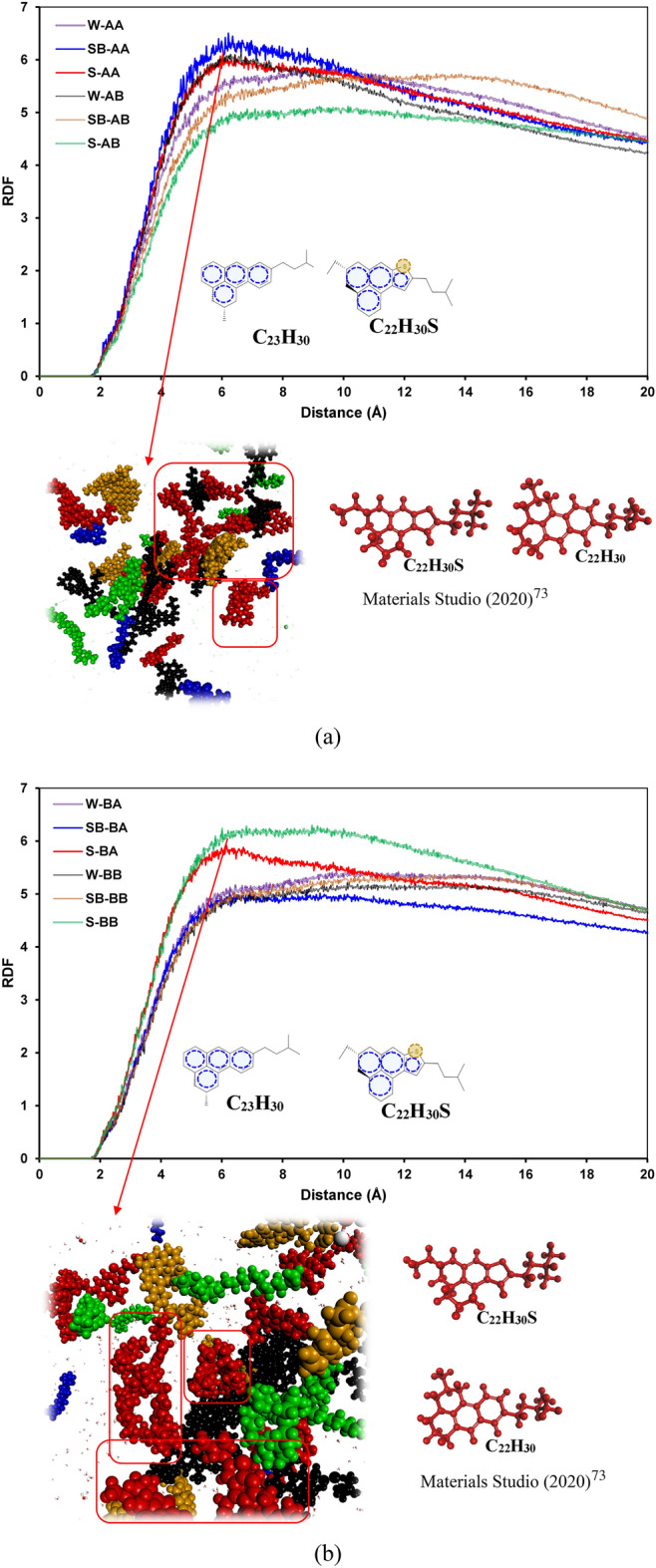
RDFs of the resin–resin molecule pairs containing (**a**) C_40_H_30_O_2_ and (**b**) C_44_H_40_N_2_OS.

Figure 8 demonstrates the RDF curves of resin–resin pair molecules for different systems. The main interaction driving forces are π–π and hydrophobic interactions between aromatic rings and hydrophobic side chains based on the resin structure. That is why we have seen spikes around 4–6 Å radial distance inside the resin pairs, representing these interactions. As demonstrated in Fig. 8a and b, systems SB-AA and S-BB have slightly higher RDF_max_ than other systems, revealing marginally greater interactions between resin pairs in these systems.

**Figure 9:**
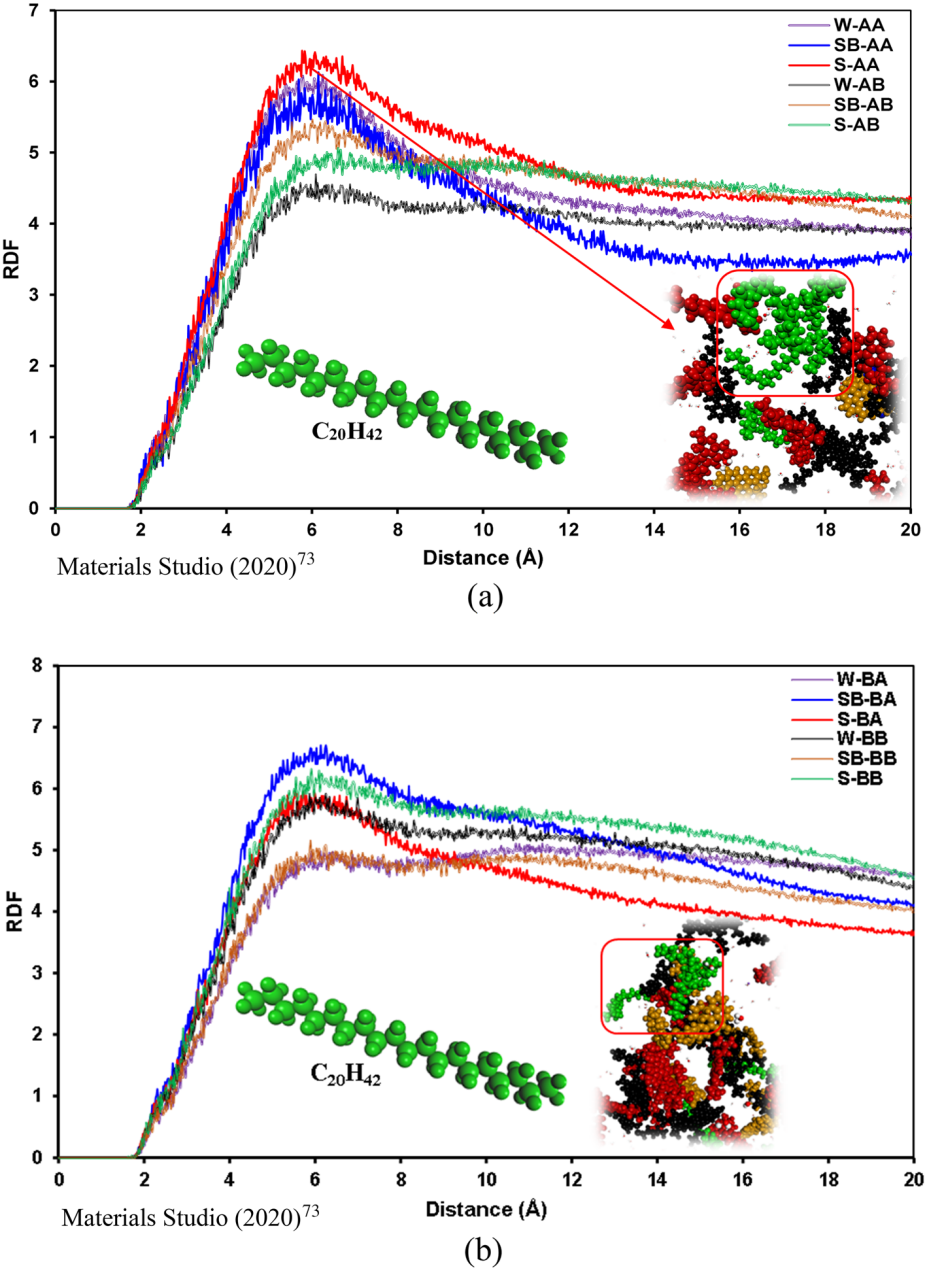
RDF of the saturate–saturate molecule pairs containing (**a**) C_40_H_30_O_2_ and (**b**) C_44_H_40_N_2_OS.

Figure 9 illustrates the RDF plots of saturate–saturate pair molecules for all systems. As depicted in Fig. 9a and b, there are spikes around 5Å distance between saturate pairs, which stand for the hydrophobic interaction between saturate molecules^101^. The RDF plots of saturate pairs for all systems follow a similar pattern. However, in the case of island asphaltenes, the lowest RDF plot was observed for system W-AB, and in the case of archipelago asphaltenes, system W-BA has the lowest RDF_max_ around 5Å. This revealed that the interaction between saturates in systems W-AB and W-BA is somewhat lower than that in other systems.

**Figure 10:**
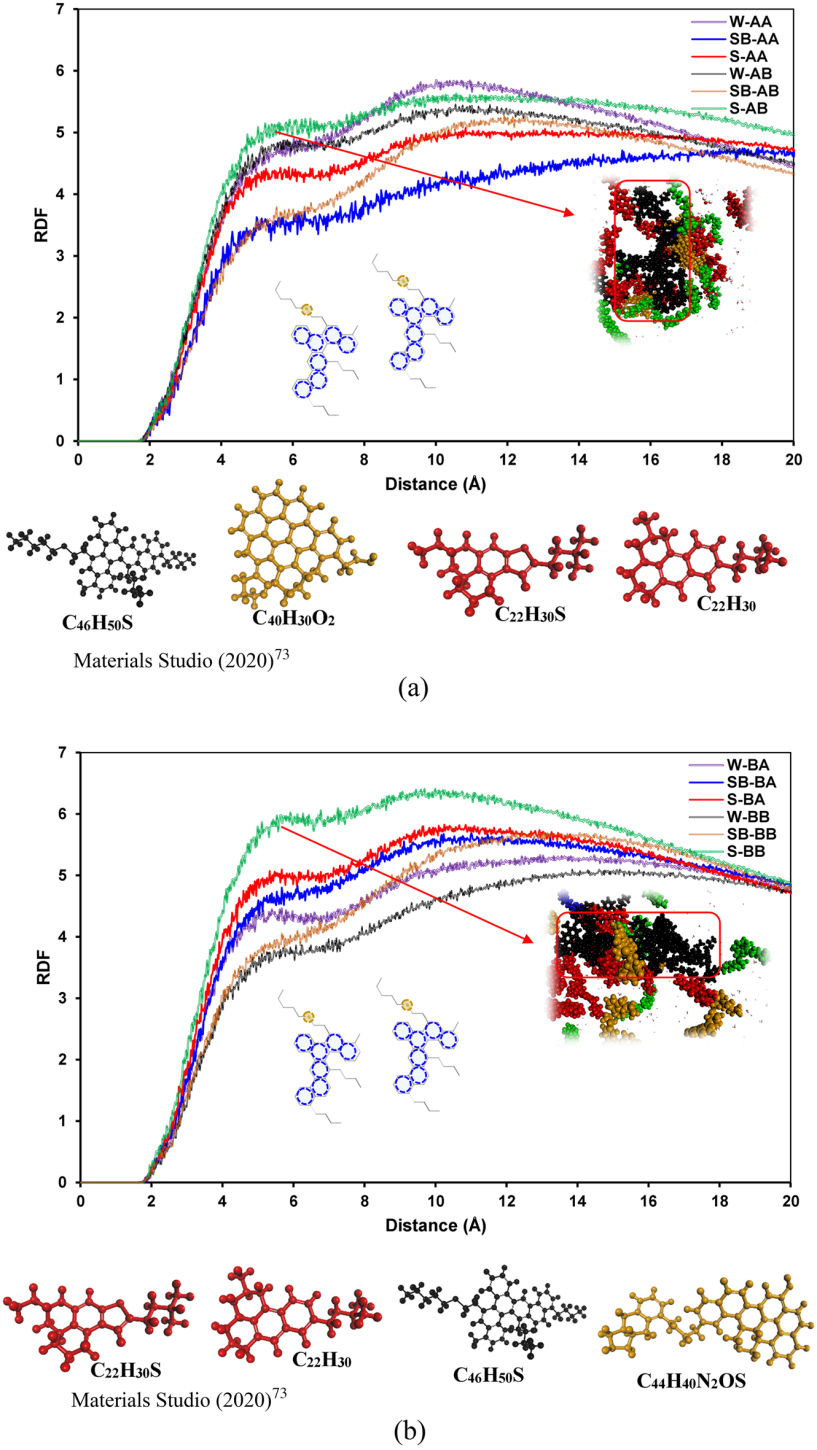
RDF of the aromatic–aromatic molecule pairs containing (**a**) C_40_H_30_O_2_ and (**b**) C_44_H_40_N_2_OS.

Figure 10 shows the RDF curves of aromatic–aromatic pair molecules for different systems. As shown in this figure, the RDF plots for aromatic pairs in different systems have a similar trend; however, aromatic pairs in systems comprised of SDS and resin B (C_22_H_30_S), for either archipelago or island architectures, have slightly greater interactions than other systems. In systems without surfactant, similar behavior was observed, although marginally higher RDFs were detected for systems having resin B (C_22_H_30_S).

### Radius of gyration

**Figure 11:**
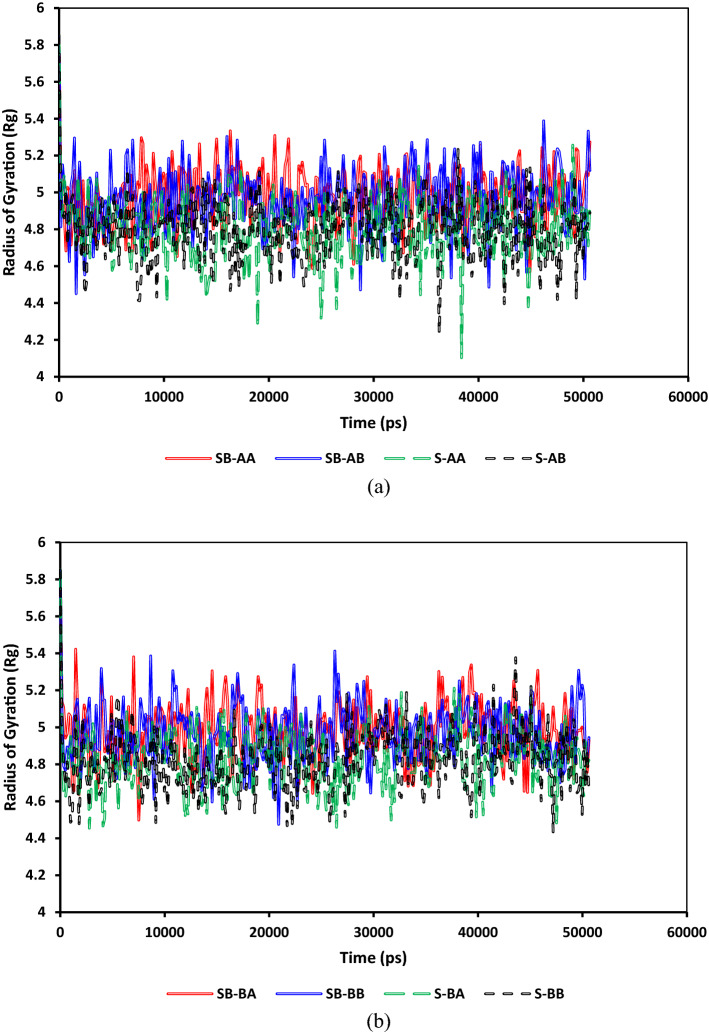
Comparison between the radii of gyration of anionic surfactants in systems containing (**a**) C_40_H_30_O_2_ and (**b**) C_44_H_40_N_2_OS.

One useful index to represent the structure and orientation of medium to large molecules, especially at the desired interface, is measuring an end-to-end distance^102^. However, this index may not be a good representative of curliness/orientation in some cases. To resolve this issue, the concept of the radius of gyration (R_g_) was introduced to provide a precise estimation of large molecules, e.g., a polymer coil size^103,104^. This concept is also applicable in a wide range of molecules such as asphaltene, surfactant, and resin^105–107^. The radius of gyration in the case of having a constant moment of inertia stands for a distance from a center of mass of a body where all the molecules can be accumulated^107^. The radius of gyration of a molecule represents a quantitative index of its stretchability. The radius of gyration (*R*_g_) can be formulated as follows^108,109^:

2 $${R}_{g}^{2}=\frac{1}{N}\sum_{j=1}^{N}{({r}_{j}-{r}_{com})}^{2}$$

where *r*_*k*_ stands for the position vector of atom *j* and *r*_com_ represents the position vector of the center of mass of a molecule^108,110^. Figure 11 compares the radius of gyration evolution for both SDS and SDBS for different systems. As depicted in Fig. 11a and b, the average radii of gyrations for SDS and SDBS are around 4.8 and 5, respectively; it does not matter which asphaltene molecules exist in a system. As illustrated in Fig. 11a and b, the gyration radii of SDBS in the cases of having island or archipelago asphaltenes are bigger than those of the corresponding systems containing SDS. This is mainly because SDBS has lower eccentricity than SDS, resulting in more flexibility in an aqueous solution, as explained by Palazzesi et al.^111^, Wei et al.^112^, and Tang et al.^113^. Moreover, as shown in Fig. 11, having sulfur in the resin structure does have a meaningful and significant effect on the compactness of surfactants at a water–oil interface.

**Figure 12:**
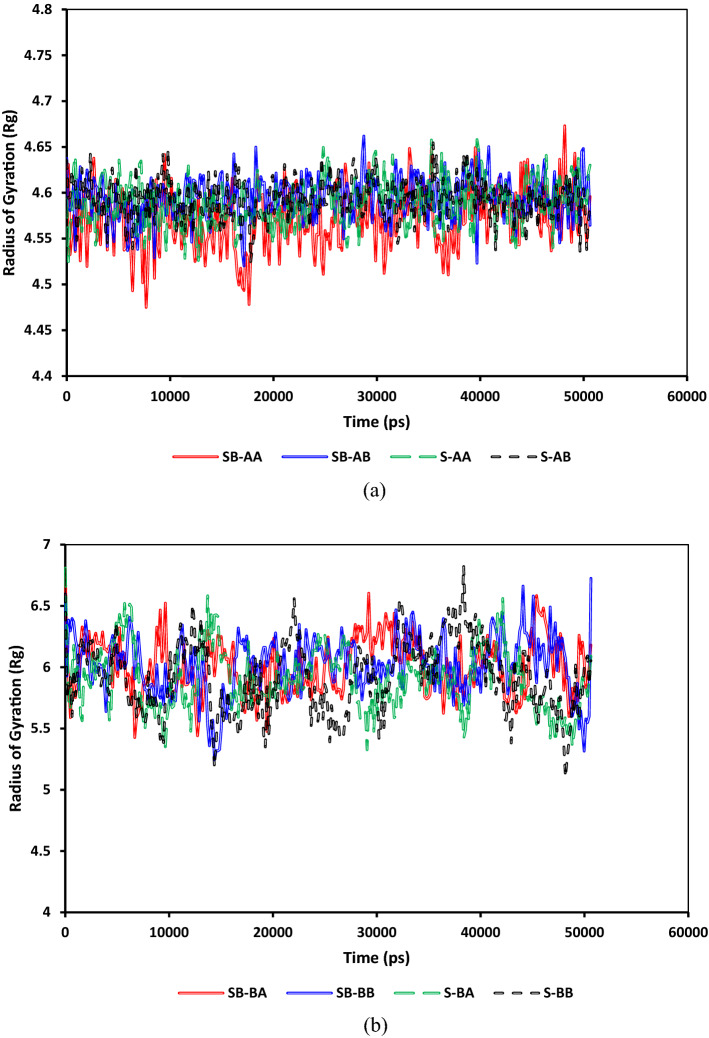
Comparison between the radii of gyration of (**a**) C_40_H_30_O_2_ and (**b**) C_44_H_40_N_2_OS.

**Figure 13:**
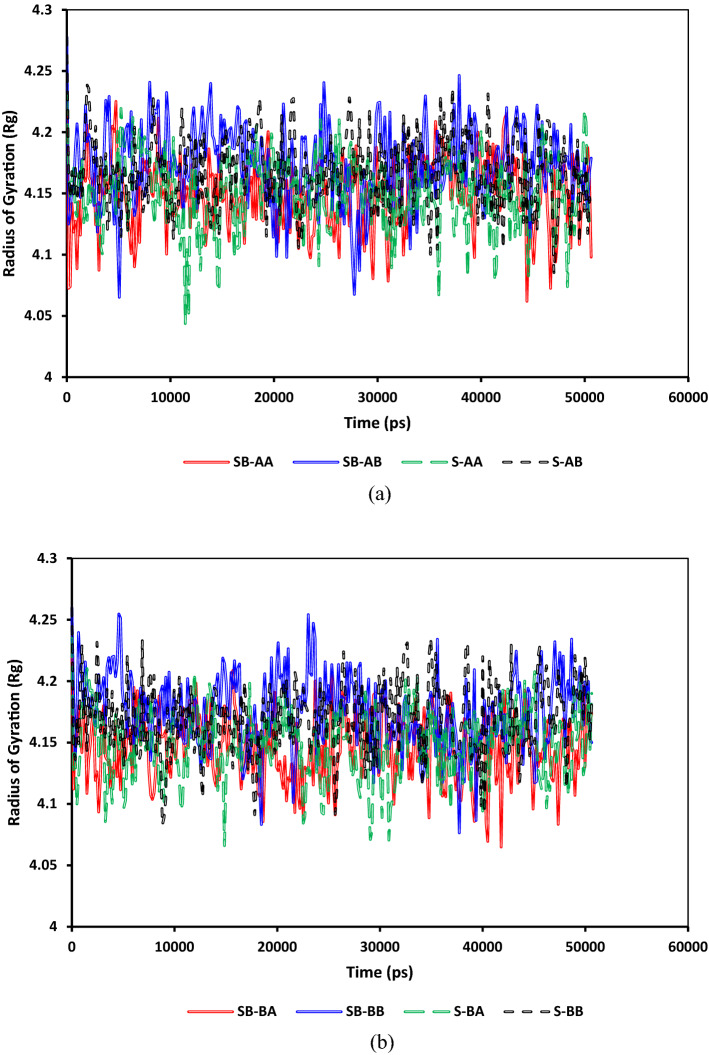
Comparison between the radius of gyration of resin for systems containing (**a**) C_40_H_30_O_2_ and (**b**) C_44_H_40_N_2_OS.

Figure 12 depicts a comparison of the radii of gyration evolution for both C_40_H_30_O_2_ and C_44_H_40_N_2_OS asphaltenes for different systems. As shown in Fig. 12a and b, the average radii of gyrations for C_40_H_30_O_2_ and C_44_H_40_N_2_OS are around 4.6 and 6, respectively. The main difference between gyration radii for island and archipelago architectures is the archipelago structure's stretchability due to having a linking alkyl bridge between two condensed aromatic segments. This architecture makes asphaltene molecules stretch/compact more than the asphaltenes with a single aromatic segment. That is why more fluctuations were observed in the gyration radii of archipelago asphaltenes. Figure 13 compares the radii of gyration evolution for both C_22_H_30_S and C_22_H_30_ for different systems. As demonstrated in Fig. 13a and b, the average radii of gyrations for C_22_H_30_ and C_22_H_30_S are around 4.15 and 4.2, respectively. The main difference between the gyration radii for C_22_H_30_ and C_22_H_30_S structures is the slightly higher stretchability of C_22_H_30_S due to having sulfur. This architecture makes resin molecules stretch/compact more than the resin without sulfur.

### Mean squared displacement (MSD)

**Figure 14:**
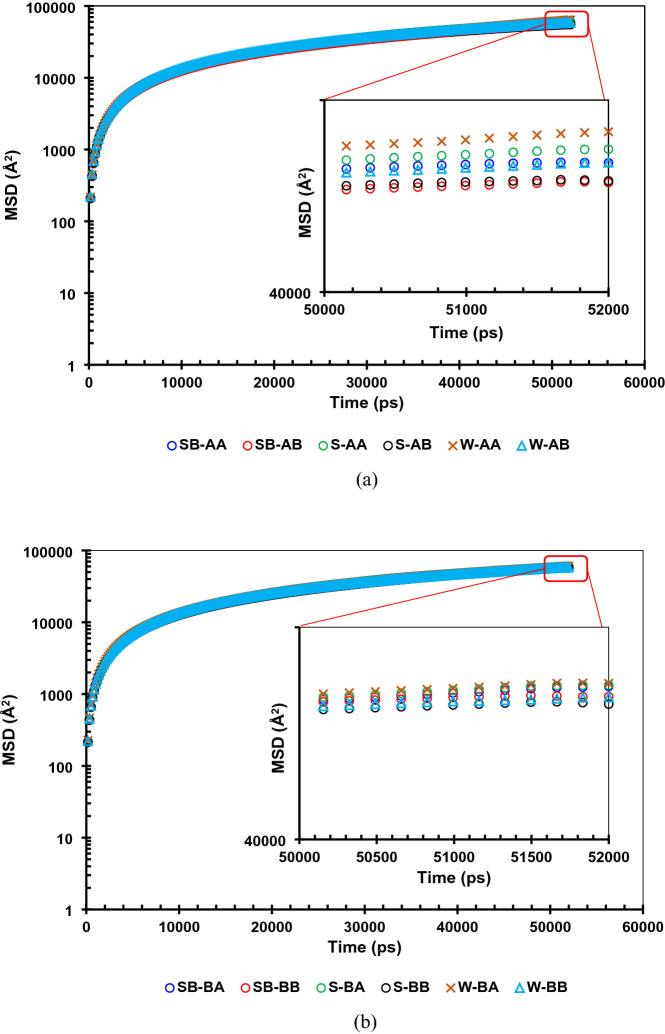
MSD of water for systems containing (**a**) C_40_H_30_O_2_ and (**b**) C_44_H_40_N_2_OS.

The mean-squared displacement (MSD) is defined by the formula below to evaluate molecules' movement and vibration during MD simulations:

3 $$MSD=\frac{1}{N}\sum_{i=1}^{N}({|\overline{{R }_{i}}\left(t\right)-\overline{{R }_{i}}\left({t}^{0}\right)|}^{2})$$

where $\overline{{R }_{i}}\left(t\right)$ represents an atom’s position as a function of time (*t*); $\overline{{R }_{i}}\left({t}^{0}\right)$ stands for an atom's initial position; *N* resembles the total number of atoms in a system. MSD curves can provide a good understanding of the behavior of the molecules in terms of displacement^114^. Figure 14 shows the MSD of water molecules versus simulation time for all systems. As illustrated in this figure, water molecules behaved similarly; however, water molecules’ movements in systems without surfactants are slightly higher than those in systems having surfactants.

### Self-diffusion coefficient (*D*)

To determine a diffusion coefficient (*D*_i_) of steam in our MD simulations, the Einstein formula^115^, as expressed below, is employed. To have a more accurate diffusion coefficient, a linear trend in MSD curves at extensive runtimes should be used^50,69^.

4 $${D}_{i}=\frac{1}{6}{\mathrm{lim}}_{t\to \infty }\frac{d}{dt} ({|\overline{{R }_{i}}\left(t\right)-\overline{{R }_{i}}\left({t}^{0}\right)|}^{2})$$

**Figure 15:**
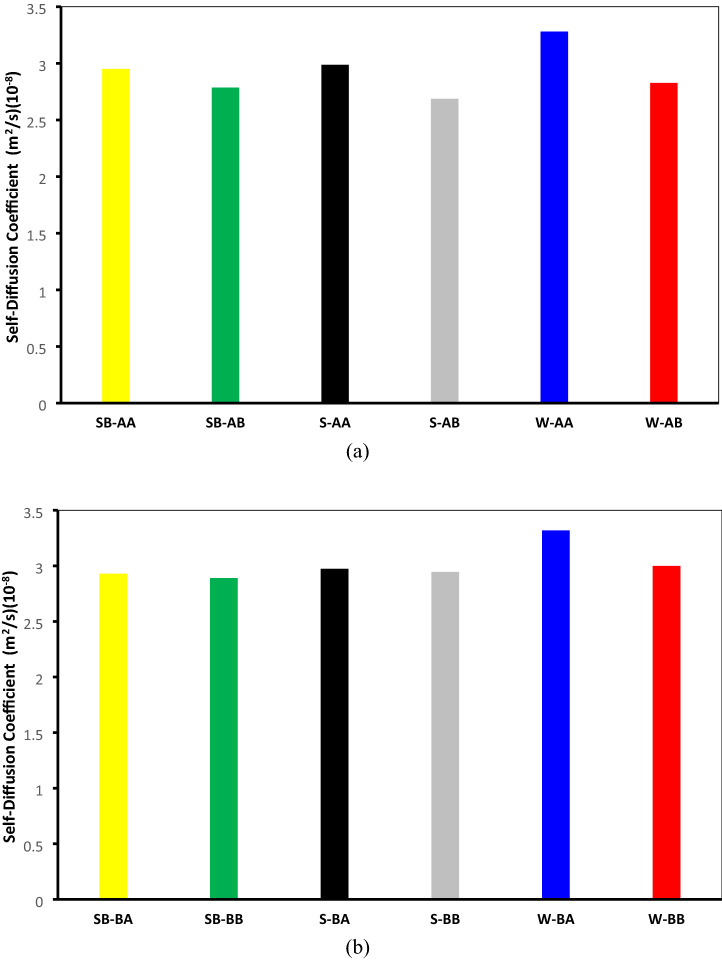
Self-diffusion coefficient of water at 498 K for systems containing (**a**) C_40_H_30_O_2_ and (**b**) C_44_H_40_N_2_OS.

A linear fit to the MSD from 10,000 to 50,000 ps and using the Einstein relation gives the water self-diffusion coefficients 2.95 × 10^–8^, 2.78 × 10^–8^, 2.98 × 10^–8^, 2.68 × 10^–8^, 3.28 × 10^–8^, and 2.82 × 10^–8^ m^2^·s^−1^ for systems SB-AA, SB-AB, S-AA, S-AB, W-AA, and W-AB, correspondingly. As shown in Fig. 15a, a water self-diffusion coefficient is decreased for systems with C_22_H_30_S. The same behavior for systems having archipelago asphaltenes was observed. With the same procedure, a linear fit to the MSD from 10,000 to 50,000 ps and using the Einstein relation gives the water self-diffusion coefficients 2.93 × 10^–8^, 2.89 × 10^–8^, 2.97 × 10^–8^, 2.94 × 10^–8^, 3.31 × 10^–8^, and 3.00 × 10^–8^ m^2^·s^−1^ for systems SB-AA, SB-AB, S-AA, S-AB, W-AA, and W-AB, correspondingly (see Fig. 15b). These results are in a reasonable agreement with the water diffusion coefficient at 498 K (3.0153 × 10^–8^ m^2^·s^−1^) reported in references^116–118^.

### Interaction energy

Interaction energy helps us examine the interaction between surfactant and heavy oi/bitumen fractions, and clarifies the strength of binding between the surfactant and different oil cuts, e.g., resin–asphaltene, resin, and asphaltene. As expressed in the equation below, the higher the absolute value, the stronger the intermolecular interaction^119^.

5 $${E}_{Inter}={E}_{Total}-({E}_{Bitumen}+{E}_{Surfactant})$$

where *E*_Inter_ stands for the interaction energy between bitumen molecules and the surfactant, kcal/mol; *E*_Total_ represents the total energy of the surfactant molecules and the bitumen system, kcal/mol; *E*_Bitumen_ denotes the energy of the bitumen system, kcal/mol; and *E*_Surfactant_ assembles the energy of the non-anionic surfactant, kcal/mol. The workflow for calculating the interaction energy is as follows^119^: (A) first, the total energy of the system, bitumen sample, and surfactant at the given temperature and pressure is calculated; (B) second, the total energy of pure substances is determined, for bitumen molecules, we must remove the surfactant molecules and then calculate the total energy, and the same is true for the surfactant molecules; (C) finally, the total energy of the entire system is deducted by the total energy of the surfactant and the total energy of bitumen molecules. The final value represents the interaction energy between a surfactant solution and oil sands/bitumen droplets. It is worth highlighting that the system geometry's optimization is required to certify the system's energy calculation stability.

**Figure 16:**
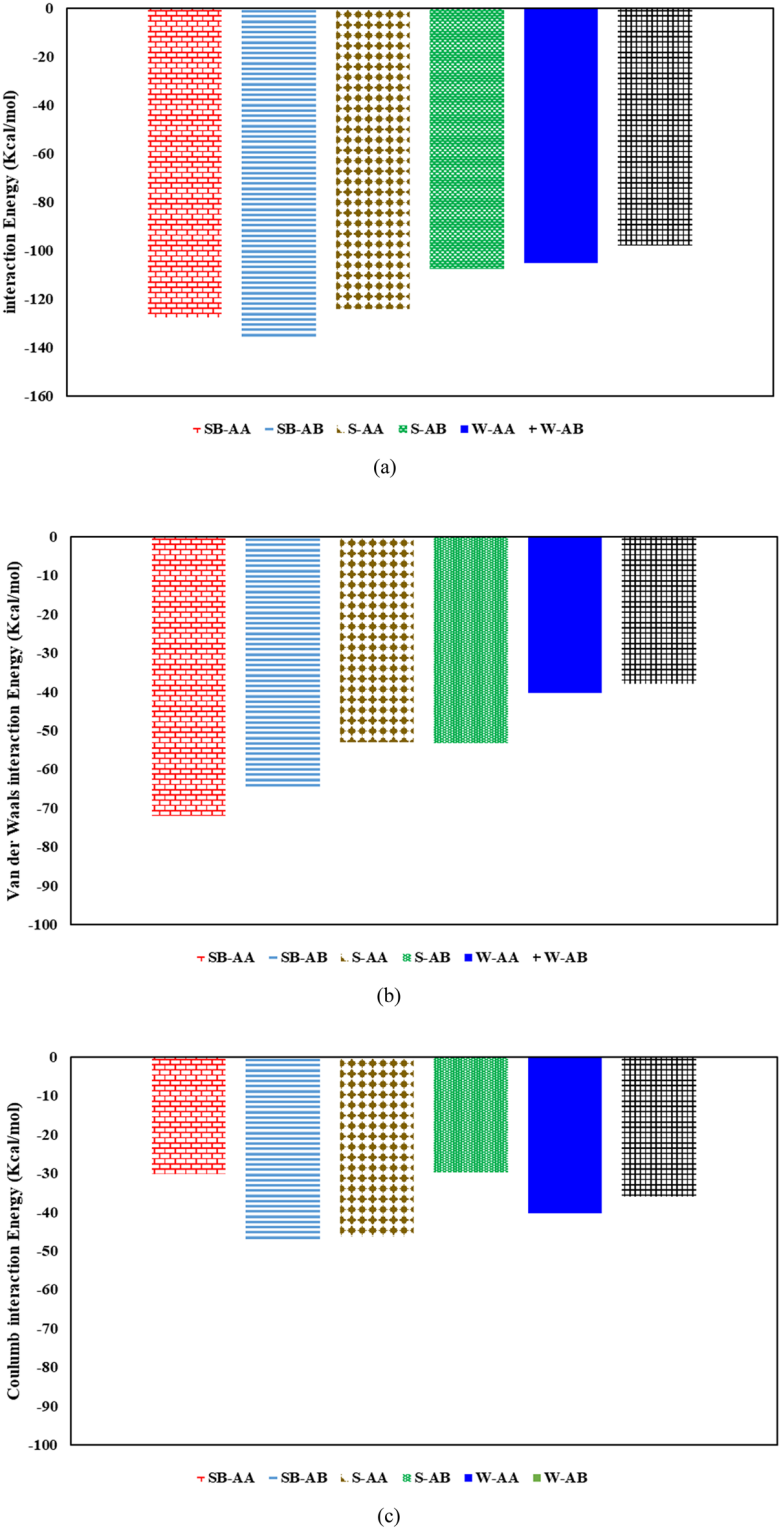
Interaction energy between surfactant molecules and bitumen containing island asphaltenes: (**a**) total interaction energy, (**b**) van der Waals interactions, and (**c**) Coulomb interactions.

Figure 16 depicts the interaction energy between surfactant solutions and bitumen droplets for island asphaltene systems. Adding surfactant molecules to the system improves the interaction energy between steam and bitumen, as shown in Fig. 16a. As demonstrated in Fig. 16a, the SB-AB system has the highest interaction energy among six different systems containing island asphaltenes. Also, this figure reveals that the systems having SDBS have higher interaction energy. In other words, for island asphaltenes, SDBS improved the interaction between an aqueous solution and bitumen droplets for having resin either A or B. Furthermore, the contribution of van der Waals interactions is higher than Coulomb interactions for systems containing surfactants.

**Figure 17:**
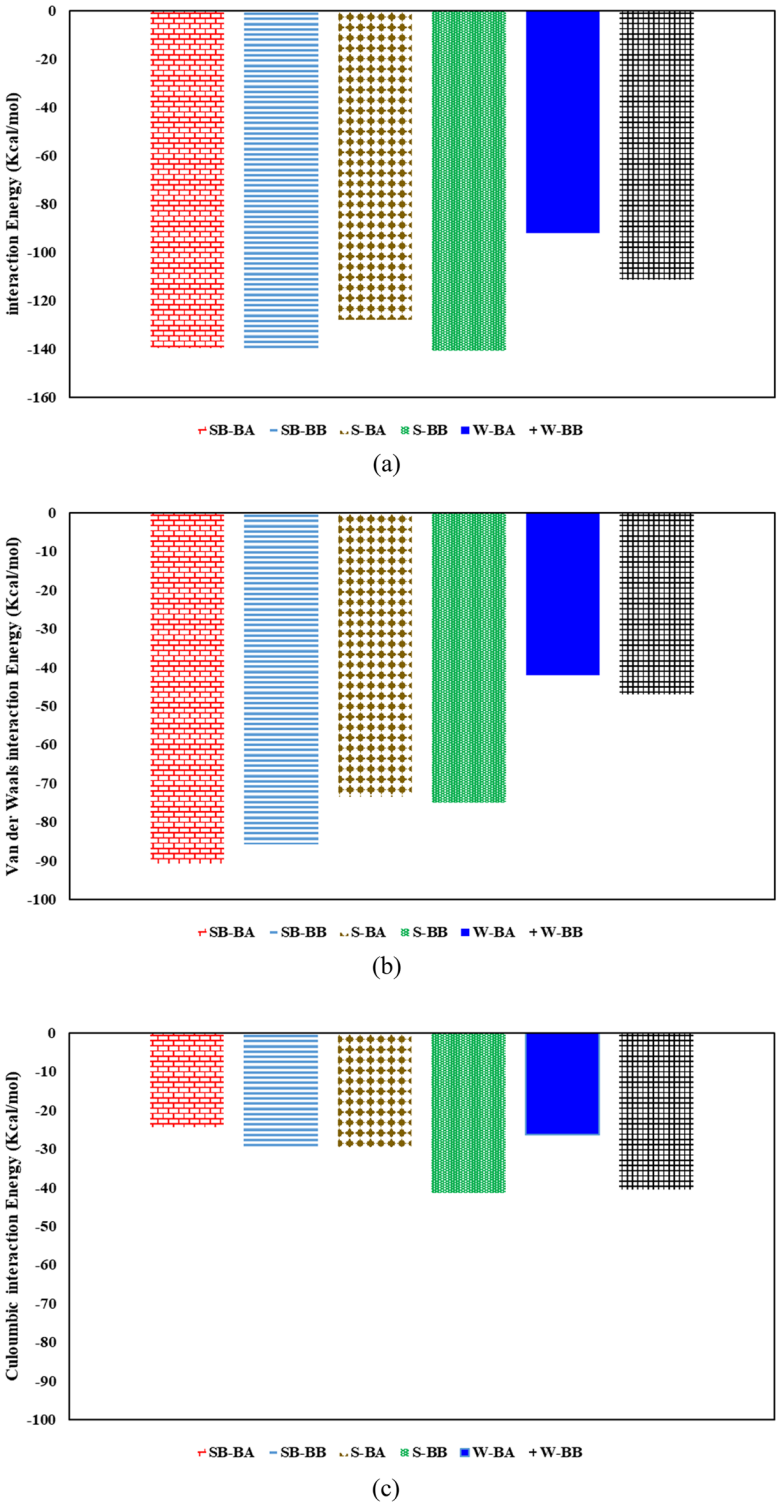
Interaction energy between surfactant molecules and bitumen containing archipelago asphaltenes: (**a**) total interaction energy, (**b**) van der Waals interactions, and (**c**) Coulomb interactions.

Figure 17 demonstrates the interaction energy between surfactant solutions and bitumen droplets for archipelago asphaltene systems. Like island asphaltenes, adding surfactant molecules to these systems increases the interaction energy between the aqueous phase and bitumen, as illustrated in Fig. 17a. As demonstrated in Fig. 17a, the interaction energy of systems containing surfactant is quite similar; however, the S-BA system has lower interaction energy than other surfactant systems. The main reason for observing such behavior is the nature of archipelago asphaltenes, which contains several heteroatoms in favor of Coulomb interactions and a large molecular area in favor of van der Waals interactions. As shown in Fig. 17b, there is no consistent behavior among systems containing C_22_H_30_ and C_22_H_30_S. In no surfactant and SDS cases, sulfur in resin resulted in higher total interaction energy, van der Waals interaction, and Coulomb interaction. Conversely, for systems with SDBS, having sulfur in resin yielded a lower van der Waals interaction and a higher coulomb interaction that maintain the total interaction energy almost constant for SB-BA and SB-BB (see Fig. 17a–c). As illustrated in Fig. 17b, the van der Waals interactions for both SB-BA and SB-BB systems are considerably high due to the interaction between benzene ring of SDBS and fused aromatic sheets of Archipelago asphaltene. Comparing Coulomb and van der Waals interactions for SB-BA and SB-BB systems reveals the resin’s sulfur heteroatom effect because the only difference between these two systems is the sulfur atom inside the resin structure. According to Fig. 17b and c, sulfur could result in lower van der Waals and higher Coulomb interactions between bitumen droplet and steam phases for SB-BA and SB-BB systems. Comparing Coulomb and van der Waals interactions for S-BA and S-BB systems revealed that both Coulomb and van der Waals interactions for S-BB are higher than S-BA due to the presence of sulfur in the resin. In other words, resin with sulfur interacted better in the system with SDS surfactant. The same trend as S-BA and S-BB was also seen for W-BA and W-BB systems.

### Solvent accessible surface area (SASA)

SASA provides an index of the available contact area between a solvent and a given molecule or aggregate. A SASA can be used as a comparison tool for different molecules or molecules with different conformations^120^. To measure the SASA of a large molecule or a cluster of molecules, a spherical probe with the radius of the solvent molecules, i.e., water, rolls over the surface of a given cluster or large molecule. The SASA of the cluster/large molecule is equal to the surface traced by the center of the spherical probe^121^. There are two well-known methods to determine a SASA, including Lee and Richards^122^ and Shrake and Rupley^123^. Lee and Richards proposed an approximation for measuring a surface by using the outline of a set of slices. Shrake and Rupley developed another method for surface measurement, which a group of test points approximates. SASA calculations have other options, e.g., an analytical method^124^ or other approximations^120,125–129^.

**Figure 18:**
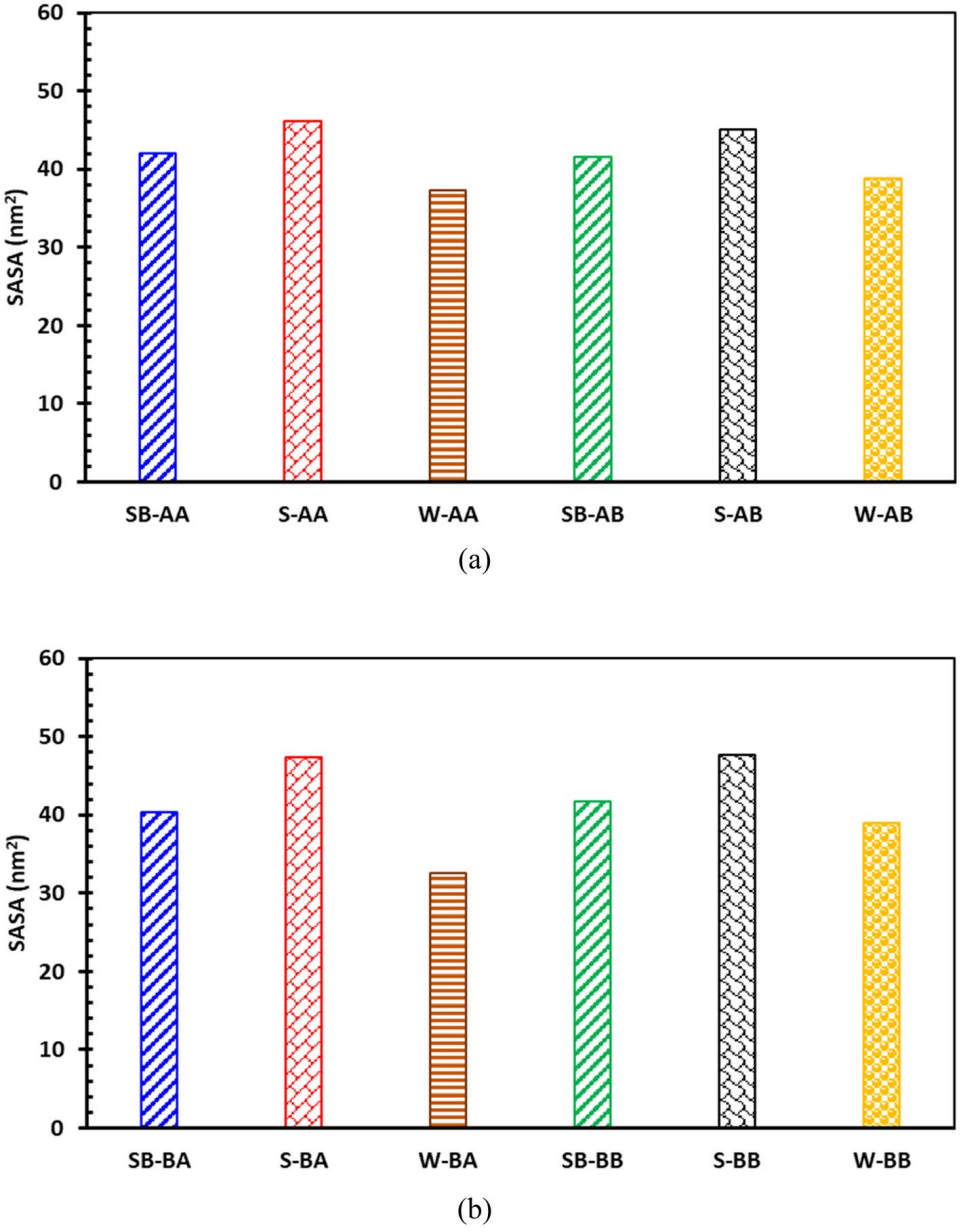
Comparison between SASAs of different systems containing (**a**) C_40_H_30_O_2_ and (**b**) C_44_H_40_N_2_OS.

Figure 18 depicts a comparison between the SASAs of bitumen droplets. As illustrated in Fig. 18a and b, the SASA for bitumen droplets containing surfactants has been greater than that for systems without surfactants. In the case of having island asphaltene (C_40_H_30_O_2_), systems containing SDS have larger solvent-accessible areas than those having SDBS. The same behavior was also observed for systems with archipelago asphaltenes. The possible reason for seeing such a trend is the capability of surfactant heads to make hydrogen bonds with water molecules. SDS has a higher capability for making hydrogen bonds than SDBS.

Furthermore, as shown in this figure, sulfur in the resin may reduce the SASA of bitumen droplets for systems with island asphaltenes and surfactants; however, it is not the case for systems without surfactants. Conversely, in systems with archipelago asphaltenes, the sulfur in the resin can increase the SASA of bitumen droplets for either having surfactant or not. The primary reason for observing this behavior is the architecture of archipelago asphaltenes and heteroatoms’ position in both asphaltene and resin molecules.

### Interfacial thickness

The “10–90” interfacial thicknesses (*t*) approach has been used to illustrate the thickness of an interface between water and bitumen throughout MD simulations. This approach is based on fitting molecular density profiles, ρ(z), of water/surfactant and bitumen to a hyperbolic tangent function as given by Eq. (6)^130^:

6 $$\rho \left(z\right)=\frac{1}{2}\left({\rho }_{L}+{\rho }_{V}\right)-\frac{1}{2}\left({\rho }_{L}-{\rho }_{V}\right)\mathrm{tanh}\left(\frac{z-{z}_{0}}{t}\right)$$

where *ρ*_L_ and *ρ*_V_ stand for the densities of liquid and vapor in the bulk phase, correspondingly, *z*_0_ represents the position of the Gibbs dividing surface and *t* denotes the interface thickness, which is defined as the space between two surfaces where the density changes from 10 to 90% of the bulk density, so *t* is called the “10–90” interfacial thickness.

Another alternative for measuring an interfacial thickness is the “10–50” interfacial thickness, which considers the density variation from 10 to 50%. Using this criterion proposed by Senapati and Berkowitz^131^ helps us calculate the thickness of a water/bitumen interface. A density profile of water and bitumen can be fitted to an error function expressed by Eqs. (7)–(8)^132^:

7 $${\rho }_{w}\left(z\right)=\frac{1}{2}{\rho }_{WB}-\frac{1}{2}{\rho }_{WB}\mathrm{erf}\left[\frac{z-{z}_{w}}{\sqrt{2}{t}_{C}}\right]$$

8 $${\rho }_{o}\left(z\right)=\frac{1}{2}{\rho }_{oB}-\frac{1}{2}{\rho }_{oB}\mathrm{erf}\left[\frac{z-{z}_{o}}{\sqrt{2}{t}_{C}}\right]$$

where ${\rho }_{w}\left(z\right)$ and ${\rho }_{o}\left(z\right)$ represent the density profiles along the *z*-direction of water and bitumen, correspondingly, ${\rho }_{WB}$ and ${\rho }_{DB}$ stand for the bulk densities of water and bitumen, individually, ⟨*Z*_w_⟩ and ⟨*Z*_o_⟩ represent the average positions of the individual Gibbs dividing surfaces for water and bitumen interface, respectively, *t*_*c*_ stands for the thickness of the interface owing to thermal fluctuation, and *erf* denotes the error function. A value of the “10−50” interfacial thickness accounts for a contribution of the thermal fluctuation (*t*_C_) and the difference between the positions of the fitted interfaces as *t*_0_ =|⟨*Z*_o_⟩ − ⟨*Z*_w_⟩| represents the contribution from the intrinsic width to the interfacial. As a result, the interface thickness *t* can be determined by Eq. (9):

9 $${t}^{2}={{t}_{0}}^{2}+{{t}_{C}}^{2}$$

**Figure 19:**
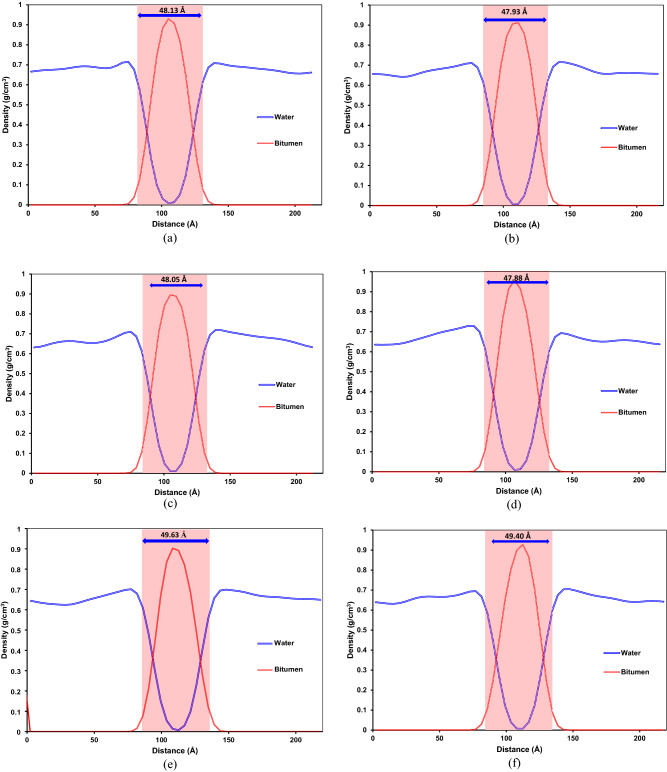

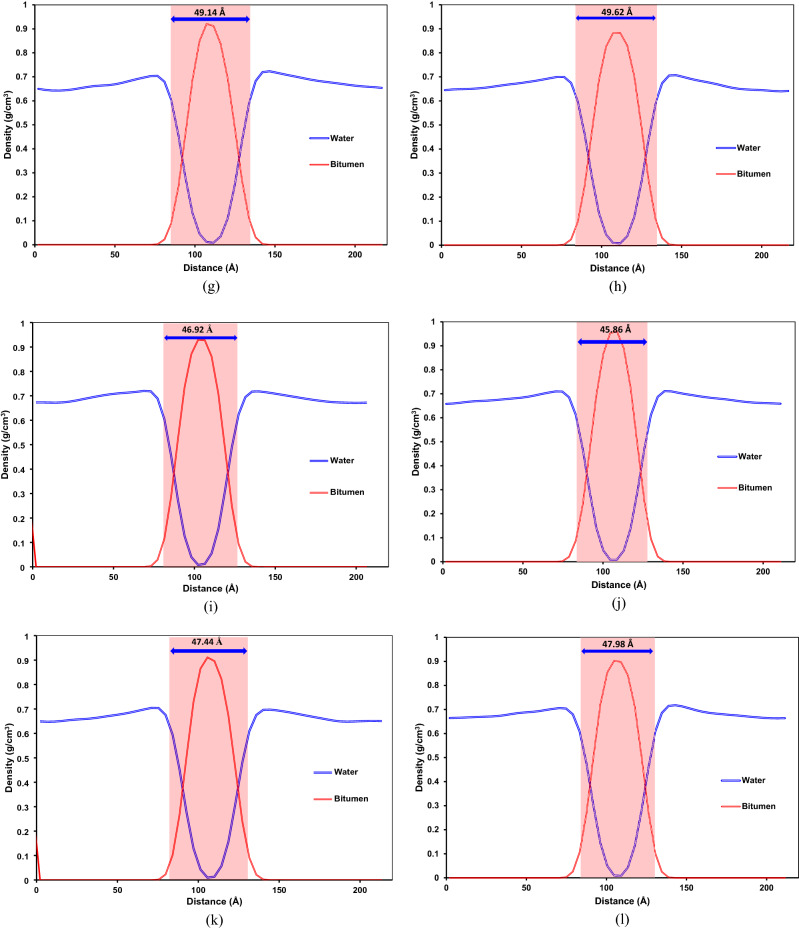
Comparison between interfacial thickness between the aqueous phase and bitumen droplets for the system: (**a**) SB-AA, (**b**) SB-AB, (**c**) S-AA, (**d**) S-AB, (**e**) SB-BA, (**f**) SB-BB, (**g**) S-BA, (**h**) S-BB, (**i**) W-AA, (**j**) W-AB, (**k**) W-BA, and (**l**) W-BB.

Figure 19 demonstrates a density profile of the steam phase and bitumen droplets in different systems and interfacial thickness profiles. As clearly seen in Fig. 19, adding a surfactant, whether SDS or SDBS, can increase the interfacial thickness between steam and bitumen droplets. Surfactants inside the aqueous phase moved toward the steam–bitumen interface and interacted with bitumen fractions, especially polar fractions, e.g., asphaltene. A higher interaction between steam and bitumen droplets resulted in a greater interfacial thickness between the two phases. It means that adding a surfactant to the steam phase improved the emulsification of the bitumen droplets into the aqueous phase. The density profile results revealed that island systems containing C_22_H_30_S have a lower interfacial thickness than those having C_22_H_30_ with similar composition. It means that bitumen droplet polar fractions in systems with C_22_H_30_S have a slightly lower interaction with the steam phase than those with C_22_H_30_.

Furthermore, due to the archipelago architecture, which contains more fused aromatic sheets and heteroatoms, greater interfacial thicknesses were observed. This is primarily because of higher interaction energies between the aqueous phase and bitumen droplets with archipelago asphaltenes. It is worth highlighting that the sulfur in resin does not have a meaningful effect on the interfacial thickness between the bitumen droplets and the aqueous solution. In other words, there is no tangible difference between the interfacial thicknesses of systems with C_22_H_30_S and C_22_H_30_ assuming similar composition in the archipelago cases.

### Interfacial tension (IFT)

The IFT between two fluids is defined as the difference between tangential and normal stresses throughout the interface. As depicted in Fig. 1, the normal axis to the interface between steam and bitumen is z-direction. Hence, the IFT can be calculated by integrating the stress difference over z-direction^132–135^:

10 $$\gamma =\frac{1}{2}\int \left({P}_{zz}-\frac{1}{2}\left({P}_{yy}+{P}_{xx}\right)\right)dz$$

where $\gamma$ stands for the IFT, P_zz_ denotes the stress along the z-direction, P_yy_ and P_xx_ represents the stress along y- and x-directions, respectively. As expressed in Eq. (11), the virial equation is used to obtain the pressure tensor for the interface system.

11 $${P}_{\alpha \beta }=\frac{1}{V}\left(\sum_{i=1}^{N}{m}_{i}{v}_{i\alpha }{v}_{i\beta }+\sum_{i=1}^{N-1}\sum_{j=i+1}^{N}{F}_{ij\alpha }{r}_{ij\beta }\right)$$

where P_αβ_ stands for αβ component in the pressure tensor, α and β represent the directional elements, V denotes the volume of the simulation box, m_i_ stands for the mass of molecule i, v_iα_ denotes the velocity of molecule i in the α-direction, F_ijα_ stands for the component α of the net force on molecule i due to molecule j, and r_ijβ_ denotes the component β of the vector (r_i_ − r_j_). The first term in Eq. (11) represents the kinetic contribution to the pressure, and the second term characterizes the virial contribution to the pressure. The required pressure elements for calculating the IFT value are three diagonal components of the pressure tensor, including P_xx_. P_yy_, and P_zz_. Integrating Eq. (10) along the length of the simulation box in z-direction yields the following equation for calculating IFT between bitumen and the aqueous phase (steam plus surfactant):

12 $$\gamma =-\frac{1}{2}\left(\frac{{P}_{x}+{P}_{y}}{2}-{P}_{z}\right){L}_{z}$$

where P_α_ = P_αα_ (α = x, y, z) and L_z_ represents the length of the simulation box along the z-direction.

**Figure 20:**
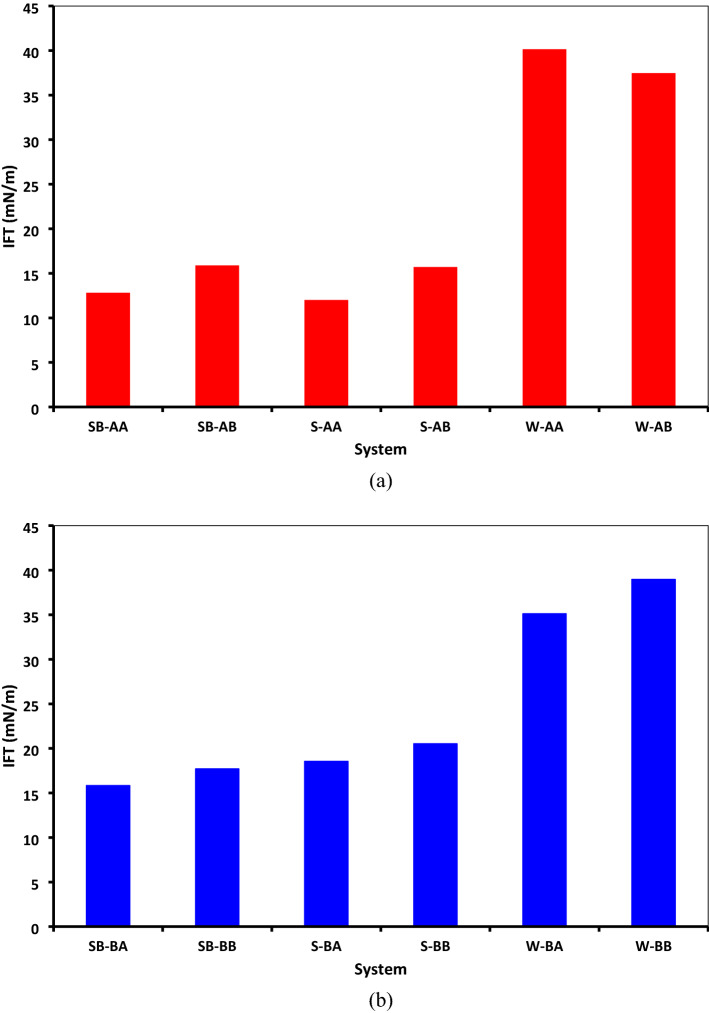
Comparison between IFT values for systems containing (**a**) C_40_H_30_O_2_, (**b**) C_44_H_40_N_2_OS.

To calculate the IFT value using the above equation, we placed surfactant molecules at the interface and followed the exact workflow described earlier. Figure 20 depicts the calculated IFT values for all systems. As illustrated in Fig. 20a, adding surfactant could significantly decrease the IFT between steam and bitumen, especially for SDBS surfactant. Comparing IFT values of systems with Island asphaltenes revealed that the presence of sulfur in resin molecules could negatively affect the IFT, resulting in higher IFT than those with sulfur. As shown in Fig. 20b, the same trend was observed for systems with Archipelago asphaltenes.

### Validation

**Figure 21:**
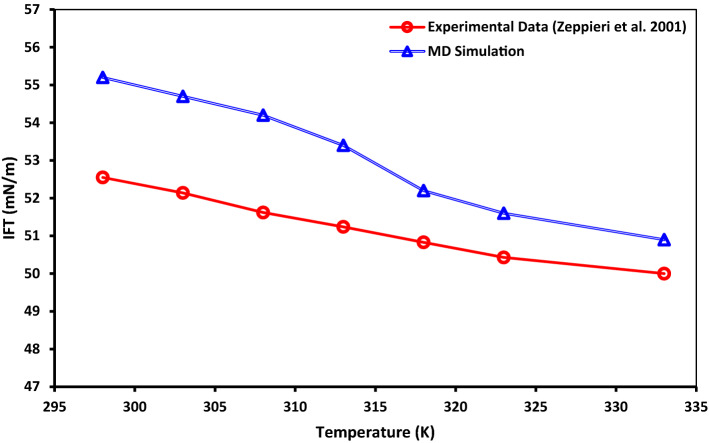
Comparison between IFT gained from experimental^136^ and MD simulation for the water–dodecane system at different temperatures.

To validate our MD simulation results, we used dodecane molecule as an oil phase and calculate water–oil IFT at different temperatures and compared the IFT values with corresponding experimental data. Two hundred fifty dodecane molecules are used as an oil phase, and the pressure of the system was set to 1 MPa, and temperature varied from 298 to 333 K. IFT value between two phases reveals the tendency of interaction between them; the higher IFT value reflects a lower tendency to interact. Figure 21 demonstrates the comparison between the IFT of the dodecane–water system gained from the experiment and MD simulation at different temperatures. As shown in this figure, increasing the temperature resulting in a lower IFT value due to increased interaction energy decreases the interfacial energy. Comparing the experimental results and MD simulation outputs for the dodecane–water system reveals that MD simulation predicts dodecane–water IFT with a reasonable amount of error. One of the probable reasons for seeing over-estimating IFT is the force-field parameters for molecules determined by empirical models or fitting experimental values. However, it should be stressed here that the COMPASS force-field parameters have been successfully developed and validated for a wide range of molecules and conditions.

## Conclusions

Interactions between surfactant, bitumen, and steam under SAGD thermodynamic conditions were thoroughly studied. A real Athabasca oil sand composition was employed to assess the phase behavior of surfactant/steam/bitumen under in-situ thermal bitumen recovery. Two different asphaltene architectures, archipelago and Island, were employed to assess the effect of an asphaltene type on bitumen's interfacial behavior. The impact of having sulfur heteroatoms in the resin structure on surfactant–steam–bitumen interactions was investigated systematically. According to the MD simulation results, the following conclusions can be drawn:

- The average radii of gyrations for SDS and SDBS are around 4.8 and 5, respectively; it does not matter which asphaltene molecules exist in a system. Gyration radii of SDBS in cases of having either Island or archipelago asphaltene are larger than those for the corresponding systems containing SDS.
- The average radii of gyrations for island and archipelago asphaltenes are around 4.6 and 6, respectively. The main difference between the gyration radii for island and archipelago architectures is the stretchability of the archipelago structure due to having a linking alkyl bridge between two condensed aromatic segments. This architecture makes the asphaltene molecules stretch/compact more than the asphaltene with a single aromatic segment.
- According to the RDF plots for asphaltene–surfactant pairs, the asphaltene architecture, resin structure, and surfactant structure significantly affect surfactant–asphaltene interactions. A benzene ring in the surfactant structure can reduce interaction between surfactant and asphaltenes no matter what architecture we have, either archipelago or Island. Moreover, having sulfur heteroatoms in the resin structure can increase interaction between SDBS and asphaltenes for archipelago and island architectures. However, resin with sulfur can significantly decrease interaction between SDS and asphaltenes for the SDS surfactant.
- Using the Einstein relation gives the water self-diffusion coefficients 2.95 × 10^–8^, 2.78 × 10^–8^, 2.98 × 10^–8^, 2.68 × 10^–8^, 3.28 × 10^–8^, 2.82 × 10^–8^, 2.93 × 10^–8^, 2.89 × 10^–8^, 2.97 × 10^–8^, 2.94 × 10^–8^, 3.31 × 10^–8^, and 3.00 × 10^–8^ m^2^·s^−1^ for systems SB-AA, SB-AB, S-AA, S-AB, W-AA, W-AB, SB-AA, SB-AB, S-AA, S-AB, W-AA, and W-AB, respectively. These results are in a reasonable agreement with the water diffusion coefficient at 498 K (3.0153 × 10^–8^ m^2^·s^−1^).
- Adding surfactant molecules to island systems improves the interaction energy between steam and bitumen. A SB-AB system has the highest interaction energy among all six different systems containing island asphaltenes. Also, the systems having SDBS have higher interaction energy. In other words, for island asphaltenes, SDBS improved interaction between an aqueous solution and bitumen droplets for having resin either A or B. Furthermore, the contribution of van der Waals interactions is greater than that of coulombic interactions for systems containing surfactants.
- Like island asphaltene, adding surfactant molecules to archipelago systems increases the interaction energy between the aqueous phase and bitumen. In cases of no surfactant and SDS, the presence of C_22_H_30_S results in higher total interaction energy, van der Waals interaction, and Coulomb interaction compared to systems with C_22_H_30_. On the contrary, for systems with SDBS, C_22_H_30_S can reduce the van der Waals interaction and increase the Coulomb interaction, which resulted in having similar total interaction energy for SB-BA and SB-BB.
- SASA for bitumen droplets containing surfactants has been higher than systems without surfactants. SDS provided larger solvent-accessible areas than those having SDBS for both archipelago and Island systems due to SDS's higher hydrogen bonding capacity. In the island systems with surfactants, sulfur in the resin has a negative effect on a SASA of bitumen droplets; however, it is not the case for systems without surfactants. On the contrary, in systems with archipelago asphaltenes, the sulfur in the resin can improve the SASA of bitumen droplets in all cases.
- Adding surfactant can increase the interfacial thickness between steam and bitumen droplets in either archipelago or island architectures. In other words, having surfactant in the steam phase provided a higher capability to emulsify bitumen droplets into the aqueous phase. According to the density profile results, island systems containing C_22_H_30_S have a lower interfacial thickness than those with C_22_H_30_. However, in archipelago cases, no significant change in interfacial thickness was observed for similar systems with different resins. According to IFT calculation using MD simulation, both surfactants could significantly decrease the IFT between steam and bitumen.

## Supplementary Information

Supplementary Information.
