# Supplementary material for: Spotlight onto surfactant–steam–bitumen interfacial behavior via molecular dynamics simulation
Source: Sci Rep. 2021 Oct 4;11:19660. doi: 10.1038/s41598-021-98633-1 (PMC8490457; doi:10.1038/s41598-021-98633-1)
Supplement: Supplementary file 1 — Supplementary Information. [file 41598_2021_98633_MOESM1_ESM.docx]

**Spotlight onto Surfactant-Steam-Bitumen Interfacial Behavior via Molecular Dynamics Simulation**

**Mohammadali Ahmadi^1*^, Zhangxin Chen^1*^**

^1)^ Department of Chemical and Petroleum Engineering, Schulich School of Engineering, University of Calgary, Calgary, AB T2N1T4, Canada

^*)^ Corresponding Authors

**Supplementary Information**

Figure S1 illustrates the molecular structures of the asphaltene, saturate, resin, aromatic, and anionic surfactant molecules used in this study^1-4^.

Materials Studio (2020)^73^


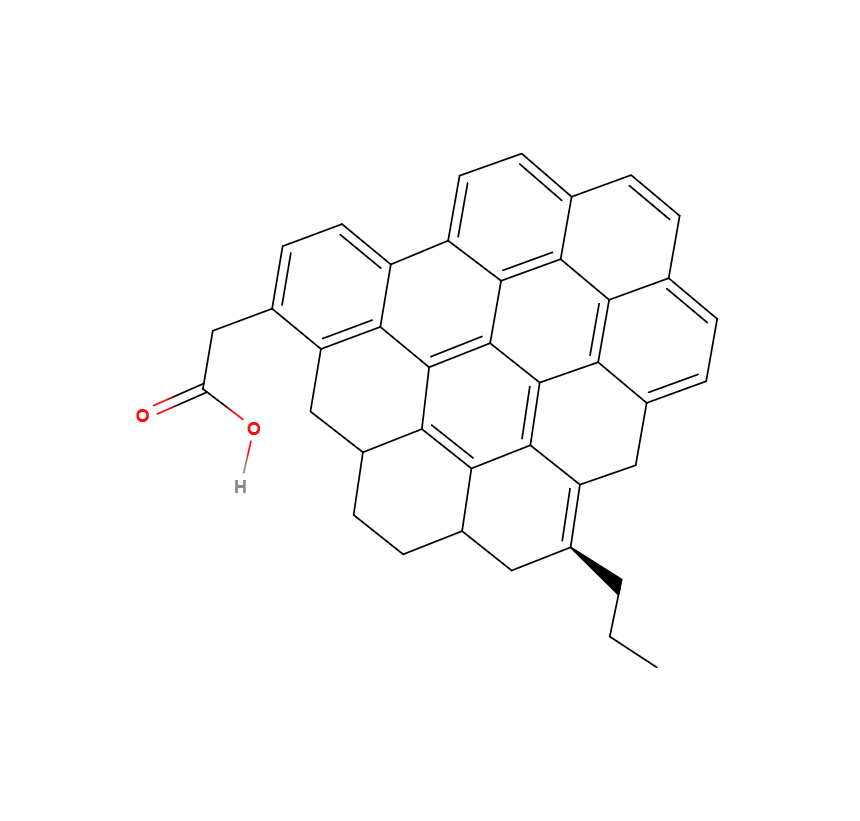


**C_40_H_30_O_2_**

**SDS**


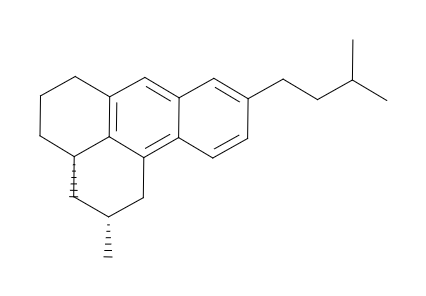


**C_23_H_30_**


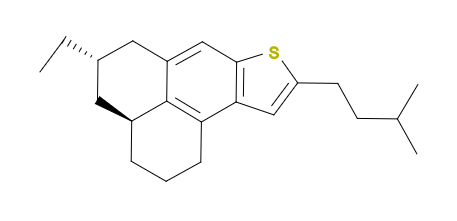


**C_22_H_30_S**

^S^


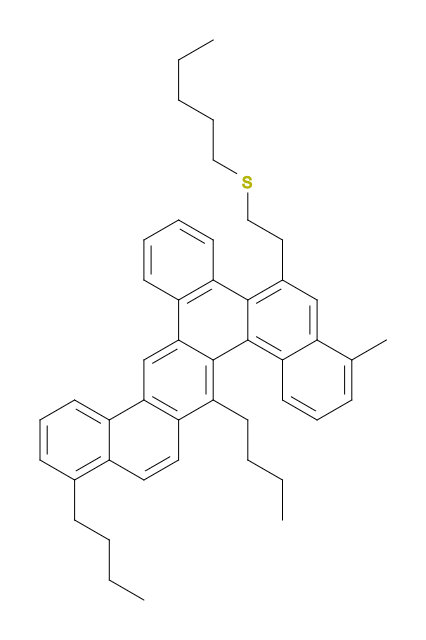


**C_46_H_50_S**

^S^


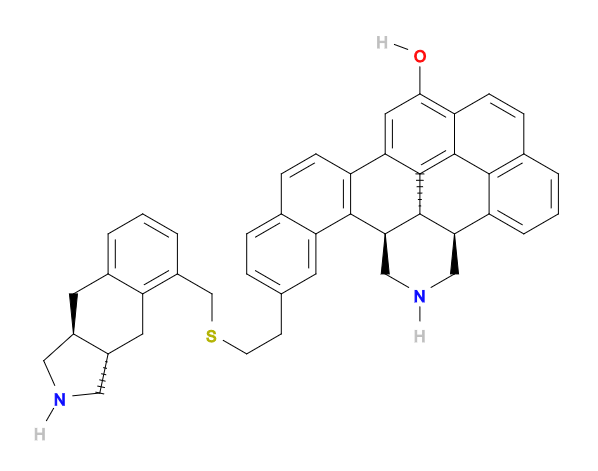


**C_44_H_40_N_2_OS**


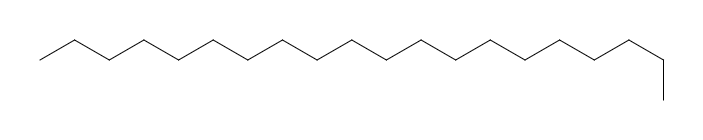


**C_20_H_42_**


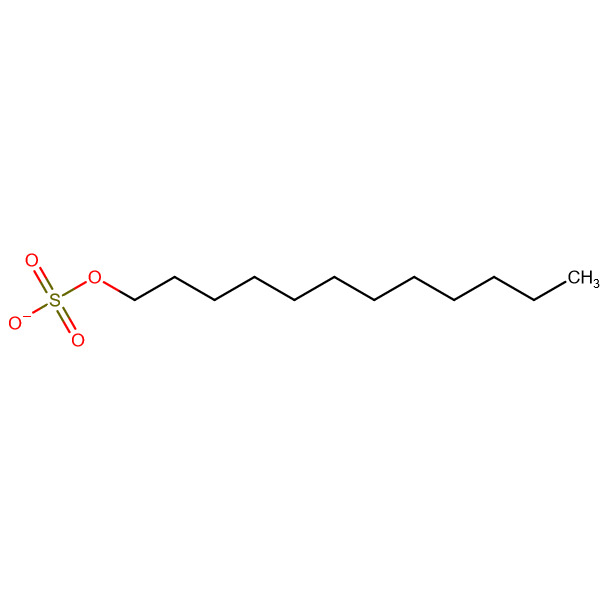


**Polar Head Group**

**Hydrophobic Tail**

**Na^+^**


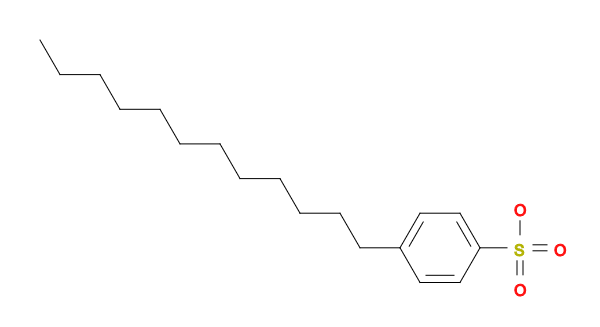


**Hydrophobic Tail**

**Polar Head Group**

**Na^+^**

-

**Benzene Connector**

**SDBS**

**Figure S1**. Chemical structures of molecules^5-7^ (blue color represents a hydrophobic part, khaki stands for sulfur, and red color denotes the polar part of molecules)

**References**

1 Yaseen, S. & Mansoori, G. A. Asphaltene aggregation due to waterflooding (A molecular dynamics study). *Journal of Petroleum Science and Engineering* **170**, 177-183 (2018).

2 Song, S. *et al.* Molecular Dynamics Study on Aggregating Behavior of Asphaltene and Resin in Emulsified Heavy Oil Droplets with Sodium Dodecyl Sulfate. *Energy & Fuels* **32**, 12383-12393 (2018).

3 Verstraete, J., Schnongs, P., Dulot, H. & Hudebine, D. Molecular reconstruction of heavy petroleum residue fractions. *Chemical Engineering Science* **65**, 304-312 (2010).

4 Wu, G., He, L. & Chen, D. Sorption and distribution of asphaltene, resin, aromatic and saturate fractions of heavy crude oil on quartz surface: molecular dynamic simulation. *Chemosphere* **92**, 1465-1471 (2013).

5 Ahmadi, M. & Chen, Z. Insight into Interfacial Behavior of Surfactants and Asphaltenes: Molecular Dynamics Simulation Study. *Energy & Fuels* (2020).

6 Ahmadi, M. & Chen, Z. Molecular Interactions between Asphaltene and Surfactants in a Hydrocarbon Solvent: Application to Asphaltene Dispersion. *Symmetry* **12**, 1767 (2020).

7 Ahmadi, M. & Chen, Z. Comprehensive molecular scale modeling of anionic surfactant-asphaltene interactions. *Fuel*, 119729, doi:<https://doi.org/10.1016/j.fuel.2020.119729> (2020).
